# The Chemical Composition and Health-Promoting Effects of the *Grewia* Species—A Systematic Review and Meta-Analysis

**DOI:** 10.3390/nu13124565

**Published:** 2021-12-20

**Authors:** Muhammad Qamar, Saeed Akhtar, Tariq Ismail, Muqeet Wahid, Ross T. Barnard, Tuba Esatbeyoglu, Zyta M. Ziora

**Affiliations:** 1 Institute of Food Science and Nutrition, Bahauddin Zakariya University, Multan 60800, Pakistan; saeedbzu@yahoo.com (S.A.); ammarbintariq@yahoo.com (T.I.); 2 Department of Food Technology, Engineering and Nutrition, Lund University, P.O. Box 188, SE-221 00 Lund, Sweden; 3 Department of Pharmacy, Bahauddin Zakariya University, Multan 60800, Pakistan; muqeetsoomro@msn.com; 4 School of Chemistry and Molecular Biosciences, University of Queensland, Brisbane, QLD 4072, Australia; rossbarnard@uq.edu.au; 5 Institute of Food Science and Human Nutrition, Gottfried Wilhelm Leibniz University Hannover, Am Kleinen Felde 30, 30167 Hannover, Germany; 6 Institute for Molecular Bioscience, University of Queensland, Brisbane, QLD 4072, Australia

**Keywords:** phytochemicals, antioxidant, inflammation, cancer, diabetes, antimicrobial

## Abstract

Globally grown and organoleptically appreciated *Grewia* species are known as sources of bioactive compounds that avert the risk of communicable and non-communicable diseases. Therefore, in recent years, the genus *Grewia* has attracted increasing scientific attention. This is the first systematic review which focusses primarily on the nutritional composition, phytochemical profile, pharmacological properties, and disease preventative role of *Grewia* species. The literature published from 1975 to 2021 was searched to retrieve relevant articles from databases such as Google Scholar, Scopus, PubMed, and Web of Science. Two independent reviewers carried out the screening, selection of articles, and data extraction. Of 815 references, 56 met our inclusion criteria. *G. asiatica* and *G. optiva* were the most frequently studied species. We found 167 chemical compounds from 12 *Grewia* species, allocated to 21 categories. Flavonoids represented 41.31% of the reported bioactive compounds, followed by protein and amino acids (10.7%), fats and fatty acids (9.58%), ash and minerals (6.58%), and non-flavonoid polyphenols (5.96%). Crude extracts, enriched with bioactive compounds, and isolated compounds from the *Grewia* species show antioxidant, anticancer, anti-inflammatory, antidiabetic, hepatoprotective/radioprotective, immunomodulatory, and sedative hypnotic potential. Moreover, antimicrobial properties, improvement in learning and memory deficits, and effectiveness against neurodegenerative ailments are also described within the reviewed article. Nowadays, the side effects of some synthetic drugs and therapies, and bottlenecks in the drug development pathway have directed the attention of researchers and pharmaceutical industries towards the development of new products that are safe, cost-effective, and readily available. However, the application of the *Grewia* species in pharmaceutical industries is still limited.

## 1. Introduction

The increasing number of deaths associated with cardiovascular disease, diabetes, cancer, inflammation, and other physiological disorders has gained the attention of health experts, researchers, and policymakers, with a view to promote healthy eating practices. Fruits and vegetables possess phytochemicals and metabolites that exhibit anticancer and anti-inflammatory effects, owing to their ability to scavenge free radicals in living systems [1,2,3]. Among these, the *Grewia* species are rich in phytochemicals and are regarded as a promising niche in averting or ameliorating the aforementioned chronic ailments. There are about 159 species of *Grewia* that are grown in tropical and sub-tropical areas of Pakistan, India, China, Malaysia, South Africa, Australia, northern Thailand, and Nigeria. Fruits of some of the *Grewia* species are edible e.g., *G. asiatica*, *G. optiva*, *G. mollis, G. occidentalis,* and *G. tenax* [4].

Numerous species of this genus have been shown to possess a variety of ethnopharmacological applications, e.g., *G. asiatica* leaves have been reported to cure skin problems such as eczema, eruptions, inflammation, as well as asthma, bronchitis, colds, coughs, and sore throat. *G. optiva* is used as “folk” medicine in the treatment of dysentery, typhoid, diarrhea, fever, cough, and smallpox [4]. *G. tiliaefolia* has been widely used to cure jaundice, biliousness, dysentery, and the diseases of the blood [5]. The ethnomedicinal formulations of *G. mollis* include infusion, decoction, maceration, or mucilage from the leaves, roots, or stem bark [6]. *G. hirsute* has been conventionally used to treat several disease conditions, such as rheumatism, joint pain, cholera, diarrhea, and ulcers [7]. *G. tenax* has been reported to cure distress of the stomach and skin, intestinal infections, fever, diarrhea, dysentery, hepatic disorders, jaundice, and rheumatism and has been reported to have antibiotic properties [8]. The boiled leaves of *G. microcos* are traditionally used to improve digestion and are also used for colds, hepatitis, diarrhea, heat stroke, dyspepsia, typhoid fever, and syphilitic ulceration of the mouth [9].

The traditional uses are increasingly supported by recent scientific research wherein some species of this genus have now been confirmed to possess anticancer, anti-inflammatory, antinociceptive, antioxidant, hepatoprotective, antidiabetic, antimicrobial, antimalarial, and sedative–hypnotic properties. They are also reported to hold immunomodulatory potential, to ameliorate learning and memory deficits, and to be effective against neurodegenerative ailments [10,11,12]. Such effects are predominantly attributed to the synergistic effects of phenolics such as flavonoids (i.e., flavones, flavanones, isoflavonoids, flavanols, dihydroflavonols, tannins, anthocyanidins), triterpenes, alkaloids, and phytosterols that are abundantly available in these species [2,13,14,15].

The *Grewia* species are also considered to be one of the most nutritious foods, since they are high in fiber, vitamins, carbohydrates, protein, and minerals, all of which are essential for a healthy lifestyle. *G. asiatica* fruits are enjoyed by people of all ages and communities in Pakistan because of their exquisite taste and affordable cost. Fresh fruits are consumed raw, and soft drinks are also produced from them. Jams, pies, squashes, and chutneys are all made using the fruit [1]. In Sudan, rural peasants utilize *G. tenax* fruits as an iron supplement for anemic children. Nesha is a thin porridge made from millet flour and the pulp of the *G. tenax* fruits, which is then thickened with custard. This porridge is provided to pregnant and breastfeeding women to help them stay healthy and produce milk for their babies [8]. In Pakistan and India, *G. optiva* fruits are edible and have a pleasant acid taste. The leaves are rated as good fodder and the trees are heavily lopped for this purpose in the winter months when no other green fodder is usually available [4].

In view of the preceding observations, a thorough and systematic analysis of the nutrients and phytochemical composition of the *Grewia* species could assist in a better understanding of the role of this genus in human nutrition and health. This review intends to provide a broad view of this species, beyond the currently available reviews, and highlights future potential biological and pharmacological research on the wide range of phytochemicals found in this genus. Herein, we systematically evaluate the nutrient and bioactive composition of the *Grewia* species, including the reported concentrations of its bioactive components and related biological activities. Additionally, a bibliometric analysis was performed for the first time, all to encourage experts from underrepresented localities of the globe to initiate new studies.

## 2. Materials and Methods

### 2.1. Literature Search and Methodology

**Figure 1:**
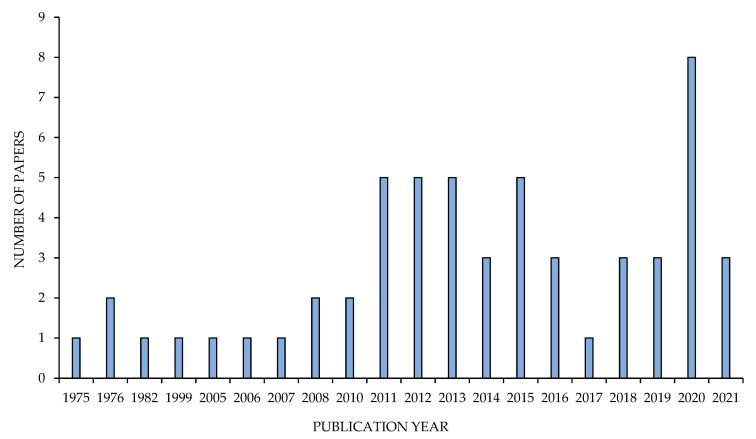
Fifty-Six articles (since 1975) retrieved with the search term “*Grewia*”. Source: PubMed, Scopus, Web of Science, Google Scholar (last accessed 31 March 2021).

The present review on the genus *Grewia* was planned and conveyed following the statement of PRISMA 10 (Supplementary S1) and based on the systematic review approach adopted by Muka et al. [16]. Different bibliographic databases (PubMed, Scopus, Web of Science, Google Scholar) were explored (Supplementary S2) to screen fifty-six relevant scientific articles published between 1975 and 31 March 2021 (date of last search) (Figure 1). The search terms were related to the nutritional aspects, and the phytochemical and pharmacological profiling of the genus *Grewia* (e.g., “nutritional composition”, “traditional medicinal uses of *Grewia*”, “biological activities of genus *Grewia*”, “phytochemical composition of *Grewia”*, “antioxidant potential of *Grewia*”, “anticancer analysis of *Grewia*”, “in vitro and in vivo anti-inflammatory activities of *Grewia*”, “anti-diabetic properties of *Grewia*”).

### 2.2. Study Selection Criteria

Articles were selected according to the criteria listed below:

i. Any parts of *Grewia* species, such as the pulp, skin, seeds, roots, bark or leaves were described;
ii. Evaluation of nutritional profiling, phytochemical composition/characterization, and pharmacological activities were provided.

Conference abstracts, letters to the editors, proceedings of conferences, literature reviews, meta-analyses, morphological studies, and product development experiments were excluded. To discover additional relevant articles, the reference lists of the included articles were checked (backward reference searching).

### 2.3. Data Extraction

**Figure 2:**
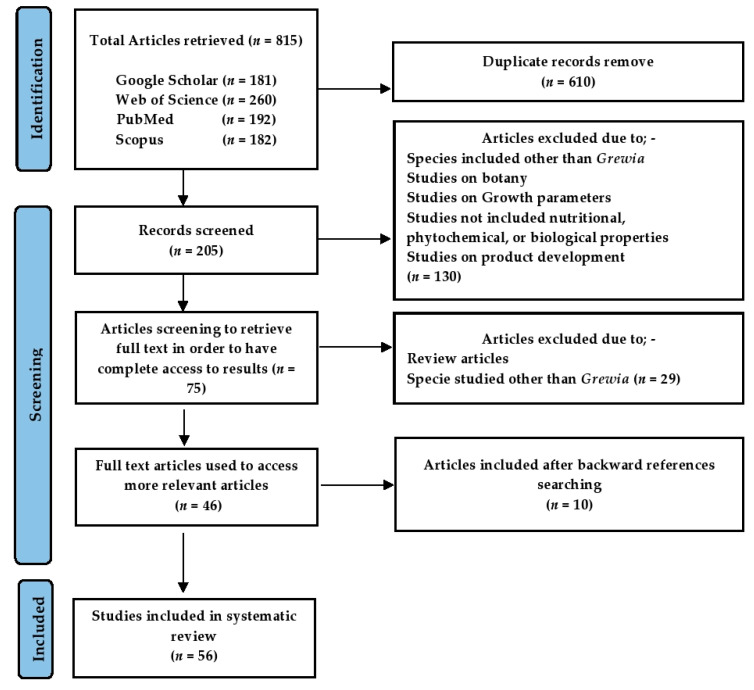
Flow diagram describing the study inclusion or exclusion criteria.

The articles were sorted or screened by two reviewers including the first and fourth authors with respect to the provided information (Figure 2).

Articles focusing on the nutritional and phytochemical composition/identification/characterization/quantification and the health-promoting impacts of the *Grewia* species, including the antioxidant, anti-inflammatory, antidiabetic, anticancer, antimicrobial and other biological activities were included in this review. The other key features were year of publication, species, type of solvent used for extraction, technique/method adopted for the identification of bioactive metabolites, and the parts of plants used in the experiment. Moreover, in vitro experiments and in vivo animal-based studies were also considered. Among all the eligible studies, eleven studies evaluated the proximate composition including carbohydrates, fat and fatty acids, protein and amino acids, fiber, ash and minerals, and vitamins. Nineteen studies evaluated the phytochemical composition including flavonoids, phenolic acids, terpenoids, phytosterols, carboxylic acid, hydroxycinnamic acid, sesquiterpenoids, hydroxycoumarins, fatty alcohol, phenols, xanthones, hydroxyquinols, and non-flavonoids. Eleven studies determined the antioxidant potential of *Grewia* using in vitro experiments. Six studies focused on in vitro anticancer properties using the 3-(4,5-dimethylthiazol-2-yl)-2,5-diphenyl-2H-tetrazolium bromide (MTT) assay against various cancer cell lines. In five articles, the anti-inflammatory properties of *Grewia* were analyzed; four of them were in vivo and one of them was in vitro. Eight studies evaluated the radioprotective/hepatoprotective potential of *Grewia* against radiation-induced thiobarbituric acid reactive substances (TBARS) and lipid peroxide production. Nine antimicrobial studies were conducted wherein four focused on antibacterial properties, two focused on antibacterial and antifungal activities, and two only studied antifungal capabilities. Seven articles evaluated antidiabetic potential wherein three studies used animal models, three used in vitro models, and one used a non-diabetic human model.

### 2.4. Bibliometric Analysis

**Figure 3:**
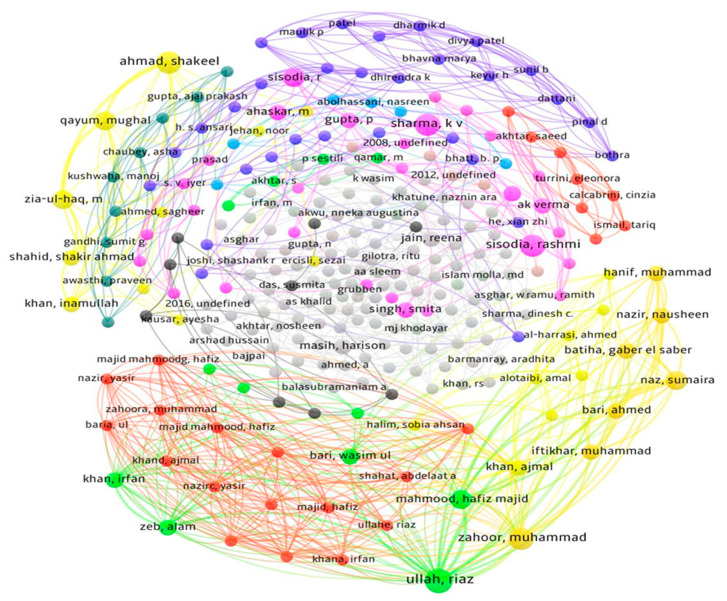
Network visualization map of the reported studies on various *Grewia* species. Clusters of different colors group authors belonging to the same region: the red, green, and yellow cluster represents authors from Pakistan, and the blue, pink, and grey cluster denotes authors from India. The few black nodes that can be seen in the picture represent South Africa and Botswana. Different clusters indicating authors from same region/country are due to the different years of publication.

Bibliometric analysis is a computational method for analyzing selected published research/review articles, as well as other related works on the subject that aims to attract experts from pharmaceutical industries paying close attention to the outcomes from this statistical method. The network maps were created based on research relationships between article authors, keywords in papers, journals in which publications are published, and organizations where research was performed (Figure 3). In the present systematic review, the analysis was performed on published data on various *Grewia* species. The co-authorship analysis was performed to investigate the interactions among scholars in relation to a research topic and a formal means for researchers to collaborate intellectually [17,18].

The goal was to create a network model that could describe the interactions between researchers from various areas of the world. For this purpose, relevant articles were found in the Mendeley database using the multiple key terms given in Section 2.1. Importantly, research articles were selected based on their publication year between 1975 and 2021 to provide the scope of research on the *Grewia* species during the last 50 years. A network visualization map was constructed based on this refined list, using VOS viewer software version 1.6.16 (available online: www.vosviewer.com, accessed on 5 November 2021) [19] for bibliometric analysis. For the study, a supported RIS file type was uploaded in the software. The type of analysis selected was “co-authors”, the unit of analysis was “authors”, the counting method was “full counting”, and the maximum number per author selected was 25. In co-authorship networks, nodes represent authors, organizations, or countries, which are connected when they share the authorship of a paper, and these insights can be used to justify and encourage new studies among experts from underrepresented localities [20].

## 3. Results

**Figure 4:**
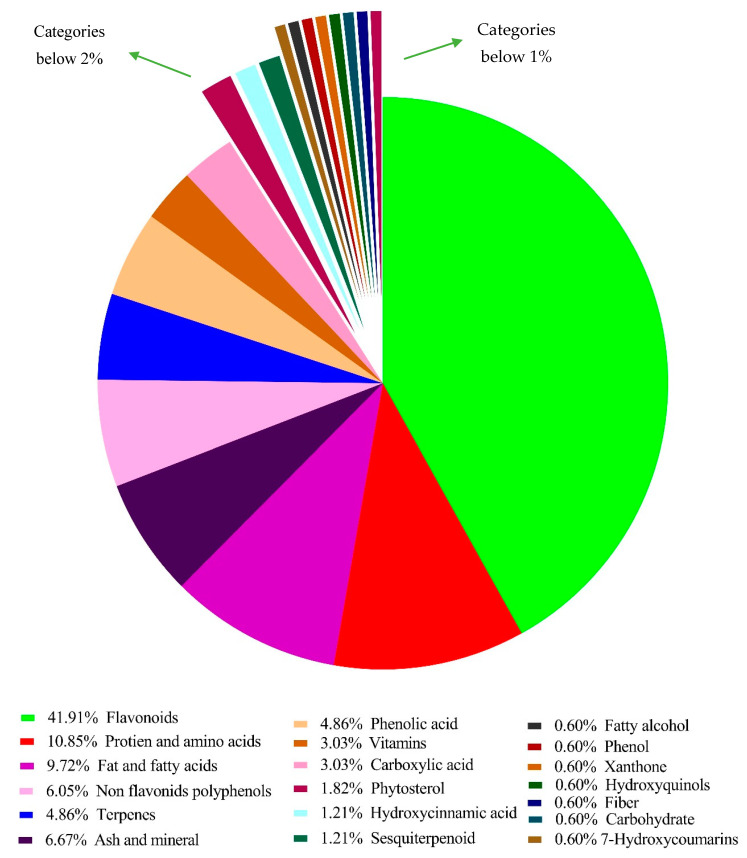
Percentage of primary and secondary metabolites in the *Grewia* species.

In general, 167 chemical compounds from 12 *Grewia* species included in the study, allocated to 21 categories were found (Table 1). Flavonoids represented 41.9% of the reported bioactive compounds, followed by protein and amino acids (10.9%), fats and fatty acids (9.72%), ash and minerals (6.67%), non-flavonoid polyphenols (6.05%), triterpenes (4.86%), phenolic acids (4.79%), vitamins (3.03%), carboxylic acids (3.03%), and all other categories were below 2% of the total reported compounds (Figure 4). Of the 167 reported compounds, information on concentrations was available for 114 (68.3%) of them, grouped in 9 categories. The information on concentration was not available for 53 compounds (31.6%) grouped in 12 categories. Moreover, Table 1 also presents the compounds according to the parts of the plant in which they were reported. A total of 15 categories were studied in fruit, 6 in seeds, 8 in leaves, 4 in stem bark, 6 in roots, and 3 in flowers.

Concerning the methods used to identify and quantify the phytochemicals, we extracted information on the solvent or extract used to analyze every compound and the techniques used to identify or quantify them. As shown in Table 1, a wide variety of extracts/solvents and techniques were reported in the literature. In detail, 25.2% of the compounds were extracted with methanol in six studies [14,21,22,23,24,25], 47.8% with acidified methanol in two studies [13,26], 11.7% with water in two studies [2,27], 5.04% with 50% methanol in one study [24], 4.20% with petroleum ether in one study [28], 3.36% with chloroform in two studies [15,29], 2.52% with ethyl acetate in one study [2], 1.68% with aqueous acetone in one study [30], and 1.68% with 80% methanol in one study [8]. Mass spectrometry was the most commonly employed technique for the identification of bioactive compounds (81.5%) wherein one study used ESI-MS/MS, one used LC–QToF–MS, one used GC-MS, and two used NMR spectroscopy. Secondly, liquid chromatography was used for the identification of bioactive compounds (11.7%), two studies employed HPLC using a diode array detector and in one article TLC was used, and information was not available for 6.70% of them.

**Table 1:** Proximate composition and the phytochemicals identified in various *Grewia* species from 1975 to 2021.

Serial Number	Primary Metabolites	Species	Plant Part	Concentration (Dry Weight)		References		
Carbohydrates								
1	Carbohydrates	*G. asiatica*	Fruits	21.1%		[31]		
1	Carbohydrates	*G. asiatica*	Leaves	29.0%		[32]		
1	Carbohydrates	*G. asiatica*	Seeds	39.7%		[33]		
1	Carbohydrates	*G. tenax*	Fruits	66.0%		[34]		
1	Carbohydrates	*G. tenax*	Leaves	28.6%		[32]		
1	Carbohydrates	*G. tenax*	Seeds	66.5%		[35]		
1	Carbohydrates	*G. flavescence*	Fruits	75.0%		[34]		
1	Carbohydrates	*G. villosa*	Fruits	84.0%		[34]		
1	Carbohydrates	*G. villosa*	Leaves	33.8%		[32]		
1	Carbohydrates	*G. tilifolia*	Leaves	40.1%		[32]		
1	Carbohydrates	*G. nervosa*	Leaves	38.6%		[32]		
Fat and fatty acids								
2	Fat	*G. asiatica*	Fruits	<0.1% (fresh weight; FW)		[31]		
2	Fat	*G. asiatica*	Leaves	2.60%		[32]		
2	Fat	*G. asiatica*	Seeds	11.1%		[33]		
2	Fat	*G. tenax*	Fruits	1.70%		[34]		
2	Fat	*G. tenax*	Leaves	3.64%		[32]		
2	Fat	*G. tenax*	Seeds	0.81%		[35]		
2	Fat	*G. flavescence*	Fruits	1.30%		[34]		
2	Fat	*G. villosa*	Fruits	1.50%		[34]		
2	Fat	*G. villosa*	Leaves	3.38%		[32]		
2	Fat	*G. tilifolia*	Leaves	3.32%		[32]		
2	Fat	*G. nervosa*	Leaves	3.86%		[32]		
3	Oleic acid	*G. asiatica*	Seeds	16.3%		[33]		
3	Oleic acid	*G. bicolor*	Seeds	19.3%		[36]		
4	Linoleic acid	*G. asiatica*	Seeds	60.1%		[33]		
4	Linoleic acid	*G. bicolor*	Seeds	53.2%		[36]		
5	Elaidic acid	*G. bicolor*	Seeds	5.70%		[36]		
6	Palmitic acid	*G. asiatica*	Seeds	12.1%		[33]		
6	Palmitic acid	*G. bicolor*	Seeds	11.4%		[36]		
7	Stearic acid	*G. asiatica*	Seeds	5.01%		[33]		
7	Stearic acid	*G. bicolor*	Seeds	5.77%		[36]		
8	Margaric acid	*G. asiatica*	Seeds	0.14%		[33]		
9	Myristic acid	*G. asiatica*	Seeds	0.41%		[33]		
10	Behenic acid	*G. asiatica*	Seeds	0.22%		[33]		
11	Linolenic acid	*G. asiatica*	Seeds	2.55%		[33]		
12	Dihydro malvalic acid	*G. asiatica*	Seeds	0.54%		[33]		
13	Dihydro sterculic acid	*G. asiatica*	Seeds	0.65%		[33]		
14	Malvalic acid	*G. asiatica*	Seeds	1.03%		[33]		
15	Sterculic acid	*G. asiatica*	Seeds	0.89%		[33]		
16	Docosanoic acid	*G. optiva*		Not evaluated		[25]		
17	Octadecadienoic acid	*G. microcos*		Not evaluated		[37]		
Protein and amino acids								
18	Protein	*G. asiatica*	Fruits	1.57% FW		[31]		
18	Protein	*G. asiatica*	Leaves	17.5%		[32]		
18	Protein	*G. asiatica*	Seeds	17.4%		[33]		
18	Protein	*G. tenax*	Fruits	7.70%		[34]		
18	Protein	*G. tenax*	Leaves	18.9%		[32]		
18	Protein	*G. tenax*	Seeds	7.50%		[35]		
18	Protein	*G. flavescence*	Fruits	8.70%		[34]		
18	Protein	*G. villosa*	Fruits	6.70%		[34]		
18	Protein	*G. villosa*	Leaves	18.8%		[32]		
18	Protein	*G. tilifolia*	Leaves	13.7%		[32]		
18	Protein	*G. nervosa*	Leaves	12.9%		[32]		
19	Aspartic acid	*G. asiatica*	Seeds	19.1%		[33]		
20	Valine	*G. asiatica*	Seeds	13.0%		[33]		
21	Leucine	*G. asiatica*	Seeds	11.0%		[33]		
22	Glutamic acid	*G. asiatica*	Seeds	11.0%		[33]		
23	Isoleucine	*G. asiatica*	Seeds	8.01%		[33]		
24	Phenylalanine	*G. asiatica*	Seeds	7.00%		[33]		
25	Threonine	*G. asiatica*	Seeds	4.06%		[33]		
26	Proline	*G. asiatica*	Seeds	3.01%		[33]		
27	Tyrosine	*G. asiatica*	Seeds	3.00%		[33]		
28	Cystine	*G. asiatica*	Seeds	1.08%		[33]		
29	Alanine	*G. asiatica*	Seeds	1.03%		[33]		
30	Arginine	*G. asiatica*	Seeds	2.07%		[33]		
31	Tryptophan	*G. asiatica*	Seeds	1.00%		[33]		
32	Lysine	*G. asiatica*	Seeds	2.00%		[33]		
33	Histidine	*G. asiatica*	Seeds	2.02%		[33]		
34	Glycine	*G. asiatica*	Seeds	1.02%		[33]		
35	Serine	*G. asiatica*	Seeds	4.02%		[33]		
Fiber								
36	Fiber	*G. asiatica*	Fruits	5.53% FW		[31]		
36	Fiber	*G. asiatica*	Leaves	38.3%		[32]		
36	Fiber	*G. asiatica*	Seeds	26.1%		[33]		
36	Fiber	*G. tenax*	Fruits	20.5%		[34]		
36	Fiber	*G. tenax*	Leaves	31.4%		[32]		
36	Fiber	*G. tenax*	Seeds	14.8%		[35]		
36	Fiber	*G. flavescence*	Fruits	42.8%		[34]		
36	Fiber	*G. villosa*	Fruits	25.5%		[34]		
36	Fiber	*G. villosa*	Leaves	28.3%		[32]		
36	Fiber	*G. tilifolia*	Leaves	29.1%		[32]		
36	Fiber	*G. nervosa*	Leaves	29.1%		[32]		
Ash and minerals								
37	Ash	*G. asiatica*	Fruits	1.10% FW		[31]		
37	Ash	*G. asiatica*	Leaves	6.30%		[32]		
37	Ash	*G. asiatica*	Seeds	5.08%		[33]		
37	Ash	*G. tenax*	Fruits	5.20%		[34]		
37	Ash	*G. tenax*	Leaves	11.4%		[32]		
37	Ash	*G. tenax*	Seeds	3.00%		[35]		
37	Ash	*G. flavescence*	Fruits	3.40%		[34]		
37	Ash	*G. villosa*	Fruits	4.00%		[34]		
37	Ash	*G. villosa*	Leaves	8.71%		[32]		
37	Ash	*G. tilifolia*	Leaves	7.96%		[32]		
37	Ash	*G. nervosa*	Leaves	8.00%		[32]		
38	Sodium	*G. asiatica*	Fruits	17.3 mg/100 g FW		[31]		
38	Sodium	*G. asiatica*	Fruits	0.41 mg/100 g		[38]		
38	Sodium	*G. asiatica*	Seeds	264 mg/100 g		[33]		
39	Potassium	*G. asiatica*	Fruits	372 mg/100 g FW		[31]		
39	Potassium	*G. asiatica*	Fruits	0.39 mg/100 g		[38]		
39	Potassium	*G. tenax*	Fruits	817 mg/100 g		[34]		
39	Potassium	*G. flavescence*	Fruits	877 mg/100 g		[34]		
39	Potassium	*G. villosa*	Fruits	966 mg/100 g		[34]		
40	Calcium	*G. asiatica*	Fruits	136 mg 100 g FW		[31]		
40	Calcium	*G. tenax*	Fruits	790 mg/100 g		[34]		
40	Calcium	*G. flavescence*	Fruits	269 mg/100 g		[34]		
40	Calcium	*G. villosa*	Fruits	536 mg/100 g		[34]		
40	Calcium	*G. asiatica*	Seeds	820 mg/100 g		[33]		
41	Phosphorus	*G. asiatica*	Fruits	24.2 mg/100 g FW		[31]		
41	Phosphorus	*G. asiatica*	Seeds	294 mg/100 g		[33]		
42	Manganese	*G. asiatica*	Fruits	1.08 mg/100 g		[38]		
42	Manganese	*G. tenax*	Fruits	5.10 mg/100 g		[34]		
42	Manganese	*G. flavescence*	Fruits	0.1 mg/100 g		[34]		
42	Manganese	*G. villosa*	Fruits	0.1 mg/100 g		[34]		
42	Manganese	*G. asiatica*	Seeds	1.03 mg/100 g		[33]		
43	Copper	*G. asiatica*	Fruits	16 µg/100 g		[39]		
43	Copper	*G. tenax*	Fruits	1.5 mg/100 g		[34]		
43	Copper	*G. flavescence*	Fruits	1.1 mg/100 g		[34]		
43	Copper	*G. villosa*	Fruits	1.2 mg/100 g		[34]		
43	Copper	*G. asiatica*	Seeds	1.09 mg/100 g		[33]		
44	Iron	*G. asiatica*	Fruits	1695 µg/100 g		[39]		
44	Iron	*G. tenax*	Fruits	20.8 mg/100 g		[34]		
44	Iron	*G. flavescence*	Fruits	26.9 mg/100 g		[34]		
44	Iron	*G. villosa*	Fruits	29.6 mg/100 g		[34]		
44	Iron	*G. asiatica*	Seeds	27.10 mg/100 g		[33]		
45	Zinc	*G. asiatica*	Fruits	58 µg/100 g		[39]		
45	Zinc	*G. tenax*	Fruits	1.9 mg/100 g		[34]		
45	Zinc	*G. flavescence*	Fruits	1.1 mg/100 g		[34]		
45	Zinc	*G. villosa*	Fruits	1.5 mg/100 g		[34]		
45	Zinc	*G. asiatica*	Seeds	2.04 mg/100 g		[33]		
46	Cobalt	*G. asiatica*	Fruits	33.0 µg/100 g		[39]		
46	Cobalt	*G. asiatica*	Fruits	0.46 mg/100 g		[38]		
47	Nickel	*G. asiatica*	Fruits	87.00 µg/100 g		[39]		
48	Chromium	*G. asiatica*	Fruits	36.00 µg/100 g		[39]		
Vitamins								
49	Vitamin B1	*G. asiatica*	Fruits	0.02 mg/100 g FW		[31]		
50	Vitamin B2	*G. asiatica*	Fruits	0.26 mg/100 g FW		[31]		
51	Vitamin B3	*G. asiatica*	Fruits	0.825 mg/100 g FW		[31]		
52	Vitamin A	*G. asiatica*	Fruits	16.1 µg/100 g FW		[31]		
52	Vitamin A	*G. asiatica*	Fruits	0.89 I.U		[38]		
53	Vitamin C	*G. asiatica*	Fruits	4.38 mg/100 g		[31]		
53	Vitamin C	*G. asiatica*	Fruits	5.21 mg/100 g		[38]		
**Secondary metabolites**								
**Serial number**	**Secondary Metabolites**	**Category**	**Species**	**Plant Part**	**Type of Extract**	**Quantity (µg/g)**	**Detection Methods**	**References**
Flavonoids								
54	Pelargonidin 3,5-diglucoside	Anthocyanin	*G. asiatica*	Fruits	Methanol	Not evaluated	Not available	[21]
55	Naringenin-7-*O*-β-D-glucoside	Flavanone	*G. asiatica*	Fruits	Methanol	Not evaluated	Not available	[21]
56	Cyanidin-3-*O*-arabinoside	Anthocyanin	*G. asiatica*	Fruit	Acidified methanol	2.29	LC-QTOF-MS/MS	[13]
57	Cyanidin-3-*O*-sambubioside	Anthocyanin	*G. asiatica*	Fruit	Acidified methanol	27.6	LC-QTOF-MS/MS	[13]
58	Cyanidin-3-*O*-(6″-malonyl-3″-glucosylglucoside)	Anthocyanin	*G. asiatica*	Fruit	Acidified methanol	1.01	LC-QTOF-MS/MS	[13]
59	Delphinidin-3-*O*-arabinoside	Anthocyanin	*G. asiatica*	Fruit	Acidified methanol	6.51	LC-QTOF-MS/MS	[13]
60	Delphinidin-3-*O*-sambubioside	Anthocyanin	*G. asiatica*	Fruit	Acidified methanol	0.80	LC-QTOF-MS/MS	[13]
61	Petunidin	Anthocyanin	*G. asiatica*	Fruit	Acidified methanol	0.40	LC-QTOF-MS/MS	[13]
62	Cyanidin-3-*O*-6″-acetylglucoside	Anthocyanin	*G. asiatica*	Fruit	Acidified methanol	695	HPLC (diode array detector)	[26]
63	Peonidin-3-*O*-6″ acetylglucoside	Anthocyanin	*G. asiatica*	Fruit	Acidified methanol	163.6	HPLC (diode array detector)	[26]
64	Pelargonidin-3-*O*-6″-acetylglucoside	Anthocyanin	*G. asiatica*	Fruit	Acidified methanol	140.4	HPLC (diode array detector)	[26]
65	Malvidin-3-*O*-glucoside	Anthocyanin	*G. asiatica*	Fruit	Acidified methanol	Traces	HPLC (diode array detector)	[26]
66	Delphinidin-3-*O*-glucoside	Anthocyanin	*G. asiatica*	Fruit	Acidified methanol	Traces	HPLC (diode array detector)	[26]
67	Peonidin-3-*O*-glucoside	Anthocyanin	*G. asiatica*	Fruit	Acidified methanol	Traces	HPLC (diode array detector)	[26]
68	Pelargonidin-3-*O*-malonyl glucoside	Anthocyanin	*G. asiatica*	Fruit	Acidified methanol	Traces	HPLC (diode array detector)	[26]
69	Calycosin	Isoflavonoid	*G. asiatica*	Fruit	Methanol	Not evaluated	LC-ESI/MS/MS	[24]
70	Dihydrodaidzein-7-*O*-glucuronide	Isoflavonoid	*G. asiatica*	Fruit	Acidified methanol	0.17	LC-QTOF-MS/MS	[13]
71	6,7,3′,4′- Tetrahydroxyisoflavone	Isoflavonoid	*G. asiatica*	Fruit	Acidified methanol	0.12	LC-QTOF-MS/MS	[13]
72	5,7,8,3′,4′- Pentahydroxyisoflavone	Isoflavonoid	*G. asiatica*	Fruit	Acidified methanol	0.51	LC-QTOF-MS/MS	[13]
73	Apigenin-6-*C*-galactoside-8-*C*-arabinoside	Flavone	*G. asiatica*	Fruit	Acidified methanol	0.71	LC-QTOF-MS/MS	[13]
74	Apigenin-7-*O*-apiosylglucoside	Flavone	*G. asiatica*	Fruit	Acidified methanol	0.33	LC-QTOF-MS/MS	[13]
75	Luteolin-4′-glucoside	Flavone	*G. asiatica*	Fruit	Acidified methanol	0.41	LC-QTOF-MS/MS	[13]
76	Luteolin-7-*O*-(2-apiosyl-6- malonyl)-glucoside	Flavone	*G. asiatica*	Fruit	Acidified methanol	20.09	LC-QTOF-MS/MS	[13]
77	Hydroxyluteolin	Flavone	*G. asiatica*	Fruit	Acidified methanol	0.23	LC-QTOF-MS/MS	[13]
78	6-Methoxyluteolin/Nepetin	Flavone	*G. asiatica*	Fruit	Acidified methanol	0.60	LC-QTOF-MS/MS	[13]
79	Genistein	Flavone	*G. asiatica*	Fruit	50% Methanol	Not evaluated	LC-ESI/MS/MS	[24]
80	Vitexin	Flavone	*G. asiatica*	Fruit	50% Methanol	Not evaluated	LC-ESI/MS/MS	[24]
80	Vitexin	Flavone	*G. tiliaefolia*	Bark	Methanol	Not evaluated	NMR	[22]
81	Isovitexin	Flavone	*G. asiatica*	Fruit	Acidified methanol	0.33	LC-QTOF-MS/MS	[13]
82	Narirutin	Flavanone	*G. asiatica*	Fruit	Acidified methanol	0.10	LC-QTOF-MS/MS	[13]
83	Hesperetin-3′-*O*-glucuronide	Flavanone	*G. asiatica*	Fruit	Acidified methanol	0.64	LC-QTOF-MS/MS	[13]
84	Naringenin	Flavanone	*G. asiatica*	Flowers	Chloroform	Not evaluated	Not available	[29]
85	Liquiritigenin	Flavanone	*G. asiatica*	Fruit	50% Methanol	Not evaluated	LC-ESI/MS/MS	[24]
86	Catechin	Flavanol	*G. asiatica*	Fruit	Acidified methanol	0.14	LC-QTOF-MS/MS	[13]
86	Catechin	Flavanol	*G. biloba*	Not mentioned	Not mentioned	Not evaluated	NMR	[40]
87	Epigallocatechin	Flavanol	*G. asiatica*	Fruit	Acidified methanol	0.23	LC-QTOF-MS/MS	[13]
88	Epigallocatechin-7-*O*-glucuronide	Flavanol	*G. asiatica*	Fruit	Acidified methanol	0.16	LC-QTOF-MS/MS	[13]
89	Epicatechin	Flavanol	*G. asiatica*	Fruit	50% Methanol	Not evaluated	LC-ESI/MS/MS	[24]
90	Kaempferol	Flavonol	*G. asiatica*	Fruit	Acidified methanol	0.87	LC-QTOF-MS/MS	[13]
90	Kaempferol	Flavonol	*G. asiatica*	Leaves	Not mentioned	Not evaluated	Not available	[41]
91	Kaempferol-3-*O*-glucoside	Flavonol	*G. asiatica*	Fruit	Acidified methanol	0.14	LC-QTOF-MS/MS	[13]
92	Kaempferol-3-*O*-xylosylglucoside	Flavonol	*G. asiatica*	Fruit	Acidified methanol	0.10	LC-QTOF-MS/MS	[13]
93	Kaempferol-3-*O*-galactoside-7-*O*-rhamnoside	Flavonol	*G. asiatica*	Fruit	Acidified methanol	2.19	LC-QTOF-MS/MS	[13]
94	Kaempferol-3-*O*-ß-D-glucorhamnoside	Flavonol	*G. asiatica*	Fruit	Acidified methanol	0.08	LC-QTOF-MS/MS	[13]
95	Methylgalangin	Flavonol	*G. asiatica*	Fruit	Acidified methanol	0.14	LC-QTOF-MS/MS	[13]
96	Methylgalangin	Flavonol	*G. asiatica*	Fruit	Acidified methanol		LC-QTOF-MS/MS	[13]
97	Myricetin	Flavonol	*G. asiatica*	Fruit	Acidified methanol	4.87	LC-QTOF-MS/MS	[13]
97	Myricetin	Flavonol	*G. asiatica*	Fruit	50% Methanol	Not evaluated	LC-ESI/MS/MS	[24]
98	Myricetin-3-*O*-arabinoside	Flavonol	*G. asiatica*	Fruit	Acidified methanol	0.11	LC-QTOF-MS/MS	[13]
99	Myricetin-3-*O*-rhamnoside	Flavonol	*G. asiatica*	Fruit	Acidified methanol	0.75	LC-QTOF-MS/MS	[13]
100	Myricetin-3-*O*-galactoside	Flavonol	*G. asiatica*	Fruit	Acidified methanol	0.73	LC-QTOF-MS/MS	[13]
101	Morin	Flavonol	*G. asiatica*	Fruit	Acidified methanol	4.25	LC-QTOF-MS/MS	[13]
101	Morin	Flavonol	*G. optiva*	Leaves	Water	Not evaluated	HPLC (diode array detector)	[27]
102	Quercetin	Flavonol	*G. asiatica*	Fruit	Acidified methanol	0.44	LC-QTOF-MS/MS	[13]
102	Quercetin	Flavonol	*G. asiatica*	Fruits	Methanol	Not evaluated	Not available	[21]
102	Quercetin	Flavonol	*G. asiatica*	Callus	80% Methanol	2.42 ng/µL	TLC	[8]
102	Quercetin	Flavonol	*G. asiatica*	Leaves	80% Methanol	4.28 ng/µL	TLC	[8]
102	Quercetin	Flavonol	*G. asiatica*	Fruit	50% Methanol	Not evaluated	LC-ESI/MS/MS	[24]
103	Quercetin-3-*O*-xyloside	Flavonol	*G. asiatica*	Fruit	Acidified methanol	5.07	LC-QTOF-MS/MS	[13]
104	Quercetin-7-*O*-glucoside	Flavonol	*G. asiatica*	Fruit	Acidified methanol	0.10	LC-QTOF-MS/MS	[13]
105	Quercetin-4′-*O*-glucoside	Flavonol	*G. asiatica*	Fruit	Acidified methanol	0.12	LC-QTOF-MS/MS	[13]
106	Quercetin-3-*O*-(6”-malonyl-glucoside)	Flavonol	*G. asiatica*	Fruit	Acidified methanol	0.31	LC-QTOF-MS/MS	[13]
107	Quercetin-3-*O*-glucosylxylosid	Flavonol	*G. asiatica*	Fruit	Acidified methanol	34.43	LC-QTOF-MS/MS	[13]
108	Quercetin-3-*O*-galactoside7-*O*-rhamnoside	Flavonol	*G. asiatica*	Fruit	Acidified methanol	0.14	LC-QTOF-MS/MS	[13]
109	Rhamnetin	Flavonol	*G. asiatica*	Fruit	Acidified methanol	2.91	LC-QTOF-MS/MS	[13]
110	Isorhamnetin-3-*O*-pentaside-7-*O*-glucoside	Flavonol	*G. asiatica*	Fruit	Acidified methanol	1.23	LC-QTOF-MS/MS	[13]
111	Quercetin 3-*O*-β-D-glucoside	Flavonol	*G. asiatica*	Fruits	Methanol	Not evaluated	Not available	[21]
112	Isorhamnetol 5-*O*- [6 “ (3-hydroxy–3-methylglutarate)]-β D-glucoside	Flavonol	*G. asiatica*	Fruits	Ethyl acetate	Not evaluated	NMR	[2]
113	Kaempferol 3-*O*-β-D-glucopyranoside	Flavonol	*G. asiatica*	Fruits	Ethyl acetate	Not evaluated	NMR	[2]
114	Kaempferol 3-*O*-β-rhamnpyrnoside	Flavonol	*G. asiatica*	Fruits	Ethyl acetate	Not evaluated	NMR	[2]
115	Quercetin 3-*O*-glucoside	Flavonol	*G. asiatica*	Fruits	Water	Not evaluated	NMR	[2]
116	Quercetin 3-*O*-rhamnoside	Flavonol	*G. asiatica*	Fruits	Water	Not evaluated	NMR	[2]
117	Quercetin 3-*O*–β-D-2–*p*-coumaroylglucoside	Flavonol	*G. asiatica*	Fruits	Water	Not evaluated	NMR	[2]
118	Myricetin 3-*O*-β-D–xyloside	Flavonol	*G. asiatica*	Fruits	Water	Not evaluated	NMR	[2]
119	Salvianolic acid D	Flavonol	*G. asiatica*	Fruit	Acidified methanol	0.40	LC-QTOF-MS/MS	[13]
120	7-Hydroxyflavan	Flavonol	*G. asiatica*	Fruit	Acidified methanol	0.10	LC-QTOF-MS/MS	[13]
121	7-*O*-Methyl cathechin	Flavanol	*G. optiva*	Root	Methanol	Not evaluated	NMR	[14]
122	Dihydroquercetin	Dihydroflavonol	*G. asiatica*	Fruit	Acidified methanol	1.03	LC-QTOF-MS/MS	[13]
123	Dihydroquercetin-3-*O*-hexoside	Dihydroflavonol	*G. asiatica*	Fruit	Acidified methanol	0.14	LC-QTOF-MS/MS	[13]
Phenolic acids								
124	Gallic acid	Phenolic acid	*G. asiatica*	Fruit	50% Methanol	Not evaluated	LC-ESI/MS/MS	[24]
124	Gallic acid	Phenolic acid	*G. asiatica*	Fruit	Methanol	Not evaluated	LC-ESI/MS/MS	[24]
124	Gallic acid	Phenolic acid	*G. optiva*	Leaves	Water	Not evaluated	HPLC (diode array detector)	[27]
125	Caffeic acid	Phenolic acid	*G. asiatica*	Fruit	Methanol	Not evaluated	LC-ESI/MS/MS	[24]
125	Caffeic acid	Phenolic acid	*G. asiatica*	Fruit	Methanol	Not evaluated	LC-ESI/MS/MS	[24]
126	Quinic acid	Phenolic acid	*G. asiatica*	Fruit	Methanol	Not evaluated	LC-ESI/MS/MS	[24]
127	Ellagic acid	Phenolic acid	*G. asiatica*	Fruit	Methanol	Not evaluated	LC-ESI/MS/MS	[24]
127	Ellagic acid	Phenolic acid	*G. asiatica*	Fruit	Methanol	Not evaluated	LC-ESI/MS/MS	[24]
127	Ellagic acid	Phenolic acid	*G. optiva*	Leaves	Water	Not evaluated	HPLC	[27]
128	Chlorogenic acid	Phenolic acid	*G. asiatica*	Fruit	Methanol	Not evaluated	LC-ESI/MS/MS	[24]
128	Chlorogenic acid	Phenolic acid	*G. asiatica*	Fruit	Methanol	Not evaluated	LC-ESI/MS/MS	[24]
128	Chlorogenic acid	Phenolic acid	*G. optiva*	Leaves	Water	Not evaluated	HPLC (diode array detector)	[27]
129	Malic acid	Phenolic acid	*G. optiva*	Leaves	Water	Not evaluated	HPLC (diode array detector)	[27]
130	Ascorbic acid	Phenolic acid	*G. optiva*	Leaves	Water	Not evaluated	HPLC (diode array detector)	[27]
131	3, 4-Dihydroxybenzoic acid	Phenolic acid	*G. asiatica*	Fruits	Water	Not evaluated	NMR	[2]
Phytosterols								
132	β-Sitosterol	Phytosterol	*G. asiatica*	Flowers	Chloroform	Not evaluated	Not available	[29]
132	β-Sitosterol	Phytosterol	*G. biloba*	Not mentioned	Not mentioned	Not evaluated	NMR	[40]
132	β-Sitosterol	Phytosterol	*G. optiva*	Root	Methanol	Not evaluated	NMR	[14]
133	Stigmasterol	Phytosterol	*G. asiatica*	Pomace	Aqueous acetone	Not evaluated	GC/MS	[30]
133	Stigmasterol	Phytosterol	*G. microcos*	Roots	Ethanol	Not evaluated	NMR	[37]
134	Campesterol	Phytosterol	*G. asiatica*	Pomace	Aqueous acetone	Not evaluated	GC/MS	[30]
Triterpenes								
135	Betulin	Triterpene	*G. asiatica*	Stem bark	Petroleum ether	Not evaluated	GC/MS	[28]
136	Lupeol	Triterpene	*G. asiatica*	Stem bark	Petroleum ether	Not evaluated	GC/MS	[28]
136	Lupeol	Triterpene	*G. lasiocarpa*	Stem bark	Chloroform	Not evaluated	GC/MS	[15]
137	Lupenone	Triterpene	*G. asiatica*	Stem bark	Petroleum ether	Not evaluated	GC/MS	[28]
138	Friedelin	Triterpene	*G. asiatica*	Stem bark	Petroleum ether	Not evaluated	GC/MS	[28]
138	Friedelin	Triterpene	*G. biloba*	Not mentioned	Not mentioned	Not evaluated	NMR	[40]
139	Epi-friedelan-3-ol	Triterpene	*G. biloba*	Not mentioned	Not mentioned	Not evaluated	NMR	[40]
140	β-Amyrin	Triterpene	*G. asiatica*	Stem bark	Petroleum ether	Not evaluated	GC/MS	[28]
141	Betulinic acid	Triterpene	*G. optiva*	Root	Methanol	Not evaluated	NMR	[14]
142	Ursolic acid	Triterpene	*G. microcos*	Root	Ethanol	Not evaluated	NMR	[37]
Hydroxycinnamic acids								
143	*p*-Coumaroyl glycolic acid	Hydroxycinnamic acid	*G. asiatica*	Fruit	Acidified methanol	0.58	LC-QTOF-MS/MS	[24]
144	5-Caffeoylquinic acid	Hydroxycinnamic acid	*G. asiatica*	Fruit	Acidified methanol	0.25	LC-QTOF-MS/MS	[24]
Carboxylic acids								
145	1,5-Dimethyl citrate	Carboxylic acid	*G. asiatica*	Fruits	Water	Not evaluated	NMR	[2]
146	Trimethyl citrate	Carboxylic acid	*G. asiatica*	Fruits	Water	Not evaluated	NMR	[2]
147	Heneicosanoic acid	Carboxylic acid	*G. biloba*	Not mentioned	Not mentioned	Not evaluated	NMR	[40]
148	Glutaric acid	Carboxylic acid	*G. optiva*	Root	Methanol	Not evaluated	NMR	[14]
149	Hexanedioic acid	Carboxylic acid	*G. optiva*	Root	Methanol	Not evaluated	NMR	[14]
Sesquiterpenoid								
150	D-Erythro-2-hexenoic acid γ-lactone	Sesquiterpenoid	*G. tiliaefolia*	*Bark*	Methanol	Not evaluated	NMR	[22]
151	Gulonic acid γ-lactone	Sesquiterpenoid	*G. tiliaefolia*	*Bark*	Methanol	Not evaluated	NMR	[22]
7-Hydroxycoumarin								
152	Umbelliferone	7-Hydroxycoumarins	*G. asiatica*	Fruit	Acidified methanol	0.10	LC-QTOF-MS/MS	[13]
Fatty alcohol								
153	Grewinol	Fatty alcohol	*G. asiatica*	Flowers	Chloroform	Not evaluated	Not available	[42]
Phenol								
154	Vidalenolone	Phenol	*G. asiatica*	Fruit	Methanol	Not evaluated	LC-ESI/MS/MS	[24]
Xanthone								
155	Mangiferin	Xanthone	*G. asiatica*	Fruit	Methanol	Not evaluated	LC-ESI/MS/MS	[24]
Hydroxyquinol								
156	1, 2, 3-Benzene triol	Hydroxyquinols	*G. optiva*	Root	Methanol	Not evaluated	NMR	[14]
Carotenoid								
157	β-carotene	Carotenoids	*G. asiatica*	Fruits	Not mentioned	0.54 µg/100 g	Not available	[38]
Other compounds								
158	5,5,7,7,11,13-Hexamethyl-2-(5-methylhexyl)icosahydro-1H-cyclopenta[a]chrysen-9-ol	Other	*G. optiva*	Stem	Methanol	Not evaluated	GC/MS	[25]
159	5-Hydroxymethylfurfural	Other	*G. asiatica*	Fruits	Water	Not evaluated	NMR	[2]
160	3,5-Dihydroxy phenyl acrylic acid	Other	*G. optiva*	Root	Methanol	Not evaluated	NMR	[14]
161	(2,5 Dihydroxy phenyl) 3’,6’,8’-trihydroxyl-4H chromen-4’-one	Other	*G. optiva*	Root	Methanol	Not evaluated	NMR	[14]
162	2,2′-(1,4-phenylene)bis(3-methylbutanoic acid	Other	*G. optiva*	Stem	Methanol	Not evaluated	NMR	[25]
163	*N*-methyl-6-β-(1′,3′,5′-trienyl)-3-β-methoxyl-3-β-methylpiperidine	Other	*G. microcos*	Roots	Ethanol	Not evaluated	NMR	[37]
164	Methanetriol mano formate	Other	*G. optiva*	Stem	Methanol	Not evaluated	GC/MS	[25]
165	Dibutyl phthalate	Other	*G. microcos*	Roots	Ethanol	Not evaluated	NMR	[37]
166	Propyl palmitate	Other	*G. biloba*	Not mentioned	Not mentioned	Not evaluated	NMR	[40]
167	(4Z, 12Z)-Cyclopentadeca-4,12-dienone	Other	*G. hirsuta*	Leaves	Methanol	Not evaluated	NMR	[23]

### 3.1. Chemical Composition

Five studies reported qualitative and quantitative analyses of the proximate composition of various *Grewia* species including *G. asiatica*, *G. tenax*, *G. flavescence, G. villosa*, *G. tilifolia,* and *G. nervosa* [31,33,34,35]. The total content of carbohydrates, fibers, lipids, proteins, and ash was reported in the fruits, seeds, and leaves. The data illustrate that carbohydrate contents were higher in the fruits, ranging between 21 and 84% [34], followed by seeds, 39–66% [33,35], and leaves, 28–40% [32]. The fat content in seeds was reported as 11.1% [33] and was almost six times higher than that recoded in fruits (0.10–1.70%) [31,34] and three times higher than leaves (2.60–3.86%) [32]. On an average basis, leaves (12.9–18.9%) and seeds (7.50–17.4%) were reported to be a rich source of protein in contrast to fruits (1.57–8.7%). A similar trend was observed for fiber wherein the leaves exhibited more fiber content, 28.3–38.3%, followed by seeds at 14.8–26.1%, and fruits at 5.53–25.5% on average. Ash content (6–11%) in leaves was on average almost three or two times higher when compared to seeds (3–5.08%) or fruits (1.1–5.2%). Table 1 represents in detail the proximate composition of the different *Grewia* species.

Fruits and vegetables contain a huge array of secondary metabolites and in fact, these metabolites form the basis for numerous commercial pharmaceutical drugs, as well as herbal remedies derived from medicinal plants [43]. Today, the pharmacological and disease-preventing role of various classes of phytochemicals is firmly established. These chemical constituents predominantly act as antioxidants, anticancer agents, detoxifying agents, and immunity-potentiating and neuropharmacological agents [2,44]. *Grewia* has been shown to contain a wide variety of phytochemicals and bioactive compounds. Among the seven *Grewia* species considered for the phytochemistry study, *G. asiatica* was explored in eleven studies, *G. optiva* in three articles, and *G. lasiocarpa, G. biloba, G. microcos, G. tiliaefolia*, and *G. hirsuta* in each study. The information on phytochemical identification/quantification was reported in 19 articles, and three of them performed the quantification analysis [8,13,26].

Regarding the plant parts analyzed in each study, the fruits of *G. asiatica* were the most explored, with five articles studying fruits alone [2,13,21,24,26,30]. Two articles focused on *G. asiatica* leaves [8,41], two explored *G. asiatica* flowers [29,42], one studied *Grewia optiva* leaves [27], one studied *G. asiatica* stems [28] and each studied *G. microcos* [37] and *G. lasiocarpa* stems [15], *G. tiliaefolia bark* [22], *G. hirsute* leaves [23], and *G. optiva* roots [14] and stems [25] to identify the phytochemical constituents.

We found 113 secondary metabolites reported from *G. asiatica*, *G. optiva*, *G. tiliaefolia, G. biloba*, *G. microcos*, *G. hirsuta*, and *G. lasiocarpa* allocated to 13 categories wherein 102 compounds were reported from *G. asiatica*, 19 were identified from *G. optiva*, three were identified from *G. tiliaefolia*, six were identified from *G. biloba,* seven were identified from *G. microcos*, one was identified from *G. hirsuta*, and one was identified from *G. lasiocarpa.* The same compounds identified in different studies were considered as a single compound with each presented with a respective reference. Flavonoids represented 41.3% of the reported bioactive compounds wherein the most dominant subgroup was anthocyanins (13.04%) followed by flavones (6.95%), flavanones (3.47%), isoflavonoids (3.47%), and flavanols (3.47%). Phenolic acids represented 6.95% of the reported compounds followed by triterpenes (6.95%), carboxylic acid (3.47%), phytosterols (2.60%), dihydroflavonols (1.73%), hydroxycinnamic acids (1.73%), sesquiterpenoids (1.73%), fatty acids (1.73%), 7-hydroxycoumarin (0.86%), fatty alcohol (0.86%), phenols (0.86%), xanthones (0.86%) and hydroxyquinols (0.86%).

Of the 113 reported secondary metabolites, information on concentration was available for only 62 (54.86%) of them, grouped in 3 categories including flavonoids (anthocyanins, isoflavonoids, flavone, flavanones, flavanols, flavonols, dihydroflavonols), hydroxycinnamic acid, and 7-hydroxycoumarins. Table 1 presents the compounds according to the parts of the plant. Eight categories of secondary metabolites were studied in fruits, four in stem bark, three in flowers, three were reported in leaves, and one in pomace. Out of 19 studies, only 3 performed the quantitative analysis [8,13,26] whereas 16 articles without quantitative information of bioactive metabolites were reported [2,14,15,21,22,23,24,25,27,28,29,30,37,40,41,42].

### 3.2. Biological Activity

#### 3.2.1. Antioxidant Activity

Antioxidant-based drug formulations are used for the prevention and adjunct treatment of complex diseases such as Alzheimer’s, stroke, cancer, diabetes, and atherosclerosis, whose etiology is partly dependent on persistent oxidative damage by free radicals. *Grewia* has been identified as a candidate for the development of nutraceutical products by virtue of an array of relevant bioactive compounds. Further investigations at the molecular level, however, are still needed to explore and discuss the mechanisms of action of these active ingredients [2,45].

Eleven studies investigated the antioxidant potential of the *Grewia* species; eight of them studied *G. asiatica* [1,24,46,47,48,49,50,51], one article focused on *G. optiva* [27], one evaluated *G. lasiocarpa* [15], and one appraised both *G. flava* and *G. biocolor* [52]. The scavenging and reducing potential of various parts of the *Grewia* species was reported to be dose dependent. 2,2-Diphenyl-1-picrylhydrazyl (DPPH) was the most commonly employed antioxidant assay utilized in nine studies along with other methods, ABTS (2,2′-azino-bis (3-ethylbenzothiazoline-6-sulfonic acid)) in three, ferric reducing antioxidant power (FRAP) in three, nitric oxide (NO) in two, and hydrogen peroxide (H_2_O_2_) in one study. The principle of the DPPH assay involves measuring the change in DPPH color from violet to pale yellow, resulting from the existence of radical scavenging compounds [53]. Six studies indicated that the *Grewia* species showed notable antioxidant potential against stable free radicals of which five studies were from *G. asiatica* [24,46,48,49,50], and one study was from *G. optiva* [27]. Among the five studies on *G. asiatica*, four studied the edible portion and one studied the leaves of *G. asiatica.* Two studies adopted aqueous methanol extraction, two studies used pure methanol, and one study used benzene for extraction. The one reported study on *G. optiva* focused on leaves and one focused on stems using methanol or water as extraction solvents. Methanol [24,49] and aqueous methanol extracts [48,50] of the *G. asiatica* fruits showed the substantial scavenging activity to be between 60 and 85%. Another study reported by Gupta et al. [46] recorded an IC_50_ of 16.19 µg/mL for the benzene extract of the *G. asiatica* leaves against free radicals in the DPPH assay, which is almost 4.8 times more than standard ascorbic acid noticed with IC_50_ 78.17 µg/mL (Table 2).

Three studies used a FRAP assay to evaluate the reducing potential of the *Grewia* species, of which two studied *G. asiatica* fruits and one studied the stem of *G. lasiocarpa* [15,24,49]. The FRAP assay estimates the electron donating capacity of any compound based on the reduction of ferric ion (Fe^3+^, as ferric tripyridyl triazine: Fe^3+^-TPTZ) into ferrous ion (Fe^2+^, as ferrous tripyridyl triazine: Fe^2+^-TPTZ) [54]. Fifty per cent of the methanolic extract of the *G. asiatica* fruit evinced a dose-dependent reducing ability of 43 mg gallic acid equivalent per gram (GAE/g) [24] which is approximately 10 times more than 100% methanolic extract of the *G. asiatica* fruit extract (4.14 mg GAE/g) [49]. Three studies used the ABTS assay to measure the antioxidant activity of the *Grewia* species. Similar to the DPPH assay, the ABTS assay determines the antioxidant activity of hydrolysates that scavenge ABTS radicals. The *Grewia* species showed a dose-dependent ABTS scavenging.

**Figure 5:**
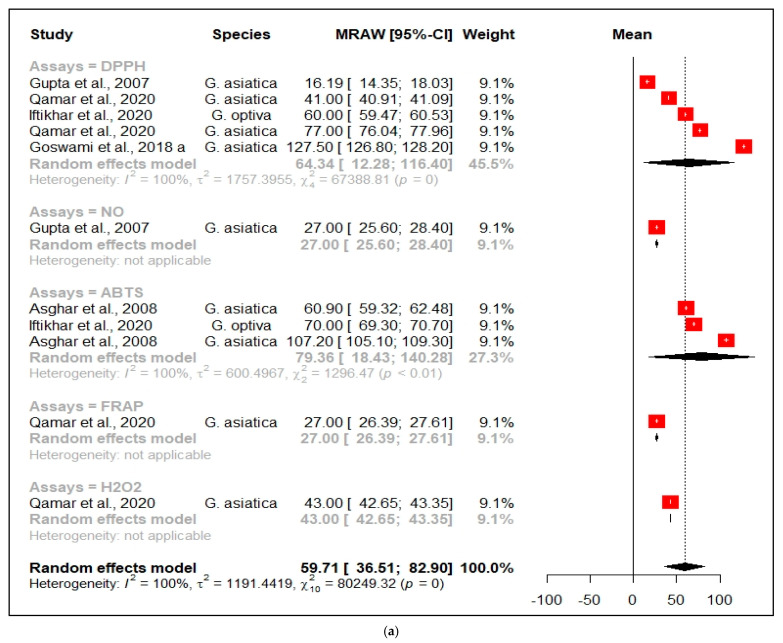

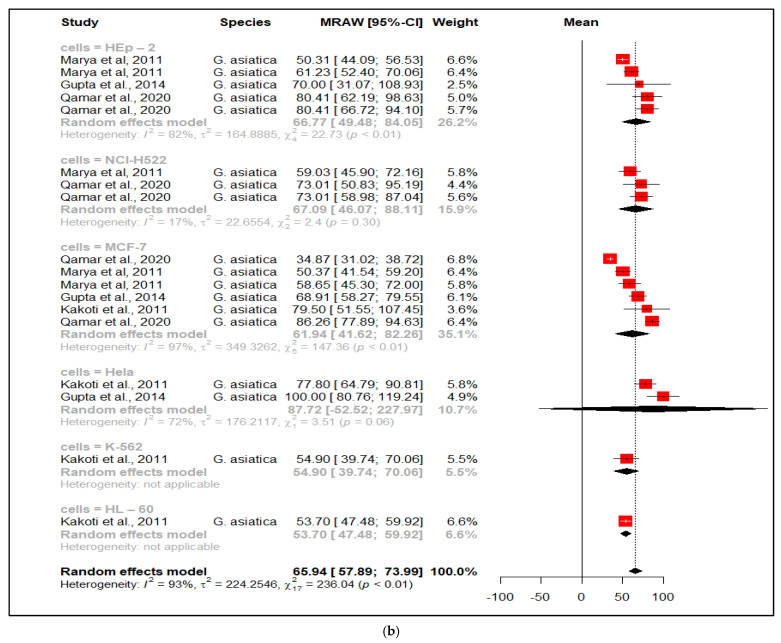

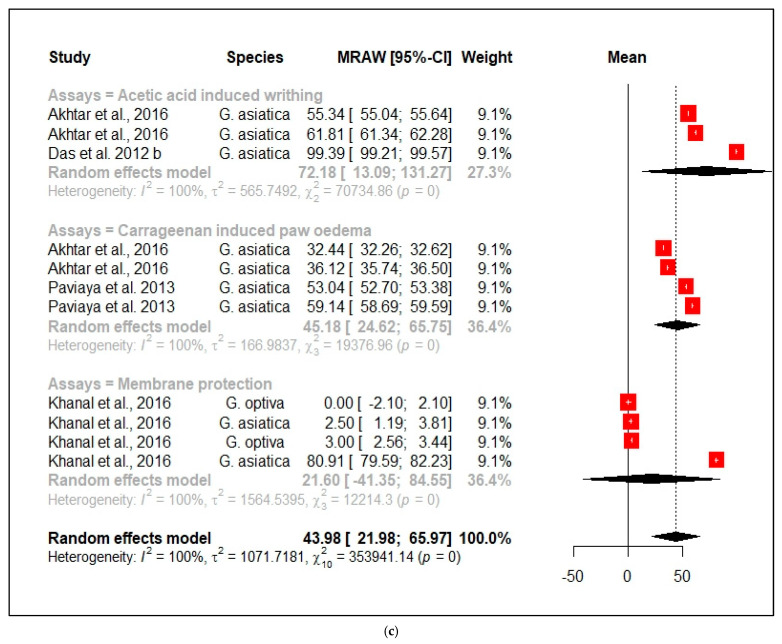

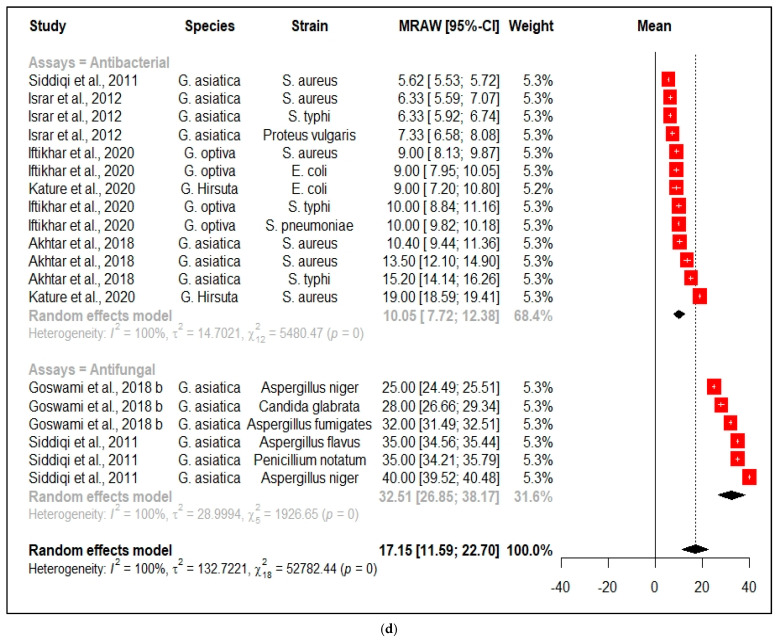
Meta−analysis of the antioxidant (**a**), anticancer (**b**), anti-inflammatory (**c**), and antimicrobial (**d**) activities [24,27,30,44,46,51,60,61,68,69,72,74,75,76,77].

In Figure 5, the meta-analysis for the antioxidant (Figure 5a), anticancer (Figure 5b), anti-inflammatory (Figure 5c), and antimicrobial (Figure 5d) activities is shown. Ten studies were included in the meta-analysis of the antioxidant activities of various *Grewia* species, as summarized in Figure 5a. The meta-analysis revealed that the *Grewia* species showed notable antioxidant activity (MRAW = 59.71, 95% CI = 36.51–82.90, *p* = 0.0, I^2^ = 100%) overall. However, a detailed sub meta-analysis of four and three studies unveiled significant antioxidant properties in the DPPH (MRAW = 64.34, 95% CI = 12.28–116.40, *p* = 0.0, I^2^ = 100%) and ABTS assay (MRAW = 79.36, 95% CI = 18.43–140.28, *p* < 0.01, I^2^ = 100%), respectively. In contrast, the NO, FRAP, and H_2_O_2_ assays were only in one study; therefore, a heterogenic analysis was not possible.

#### 3.2.2. Anticancer Properties

Despite the overwhelming research response by researchers, cancer still represents the second leading cause of death and is trending towards becoming the leading cause in the elderly [78]. Besides the tremendous development in anticancer therapies and drugs, the prevention of tumor generation by adopting a healthy lifestyle is generally considered as an effective strategy to reduce cancer risk. It is well established that diets rich in fruit and vegetables are useful in cancer prevention by virtue of their content of a wide variety of phytochemicals [79]. Their preventive activity goes beyond the antioxidant capacity, and includes effects on the expression of oncogenes, tumor suppressor genes and transcription factors, and on cell cycle and apoptosis [80,81].

Six studies were reported from 2011 to 2020 that evaluated the anticancer potential of the *Grewia* species, wherein five articles reported the anticancer effects of *G. asiatica*, one study focused on *G. lasiocarpa*, and all the studies employed the MTT assay as an index of cell proliferation. Among five studies on *G. asiatica*, two studies used a methanolic extract from the leaves, one study used the methanolic extract from fruit residues, one study used an aqueous methanol extract from fruit, one study presented the comparison between the aqueous extract from both fruits and leaves, and the last study used stem bark of *G. lasiocarpa*. In all studies, samples were prepared by initially drying the fruit/leaves/stems under shade and then were extracted with the previously mentioned solvents.

Five studies were included in the meta-analysis of the anticancer activities of various *Grewia* species, as summarized in Figure 5b, except for one study reported by Dattani et al. (2011) [56] which was excluded as the results were presented in different units. The meta-analysis revealed that the *Grewia* species showed anticancer activities (MRAW = 65.94, 95% CI = 57.89–73.99, *p* < 0.01, I^2^ = 93%) overall. However, a detailed sub-meta-analysis for specific cancer cell lines showed that *G. asiatica* exerted profound effects against the proliferation of HepG2 cells (MRAW = 66.77, 95% CI = 49.48–84.05, *p* < 0.01, I^2^ = 82%), NCI-H 522 (MRAW = 67.09, 95% CI = 46.07–8.11, *p* < 0.30, I^2^ = 70%), MCF-7 (MRAW = 61.94, 95% CI = 41.62–82.26, *p* < 0.01, I^2^ = 97%), and HeLa (MRAW = 87.72, 95% CI = −52.52–227.97, *p* < 0.06, I^2^ = 72%). In contrast, there was only one study that discussed the effects of the *G. asiatica* fruit against K562 and HL-60 (human leukemia) cells; therefore, a meta-analysis was not possible for that study.

Marya et al. [55] observed significant cytotoxic effects of the aqueous extract from the *G. asiatica* fruit against HEp-2 (larynx cancer), NCI-H522 (lung cancer), and MCF-7 (breast cancer) with IC_50_ of 50.31 µg/mL, 59.03 μg/mL and 58.65 μg/mL, respectively. However, notable activity of the aqueous extract from the *G. asiatica* leaves was observed in the aforementioned study against HEp-2 and MCF-7 cancer cell lines with IC_50_ extracts of 61.23 µg/mL, 50.37 µg/mL, respectively. Dattani et al. [56] recorded a similar response of ethanolic extracts from the *G. asiatica* fruit against NCI-H522 and MCF-7 cells while the extracts appeared to be ineffective against a cervical cancer cell line (HeLa) and HEp-2. Moreover, the intraperitoneal administration of methanolic extracts from the *G. asiatica* fruit inhibited the growth of Ehrlich’s ascites carcinoma (EAC) cells resulting in a significant increase in the life span of tumor-bearing animals and exerted cytotoxic activity toward four human cancer cells i.e., HL-60, K-562, MCF-7, and HeLa with an IC_50_ of 53.7, 54.9, 199.5, and 177.8 µg/mL, respectively [57]. With regard to the pomace extract from *G. asiatica*, this was shown to elicit a significant cytotoxic activity against MCF-7 with an IC_50_ of 68.91 µg/mL, and a less remarkable activity towards bone sarcoma cells (MG-63), HeLa, and hepatocellular carcinoma cells (HepG2) [30]. A recent study illustrated that the aqueous methanol extract from *G. asiatica* to be more effective against breast cancer, lung cancer, and laryngeal cancer cell lines with IC_50_ of 34.87 µg/mL, 73.01 µg/mL, and 80.41 µg/mL, respectively suggesting antitumor claims for the *G. asiatica* [24]. However, the stem, bark, leaves, and pulp extracts from *G. asiatica*, when analyzed for cytotoxic potential by using a brine shrimp lethality assay and a hemagglutination assay, failed to show a significant cytotoxic response [82]. The last study [15] reported anticancer effects of the pure compound lupeol i.e., isolated from the stem bark of *G. lasiocarpa* against HEK293 (human embryonic kidney), HeLa, and MCF-7 cells.

#### 3.2.3. Anti-Inflammatory Activity

The therapeutic role of medicinal plants alone or as adjuncts to conventional treatments, is firmly recognized. This notion, along with the relatively low cost of medicinal plants, has been a reason to promote their use in poor countries where people have restricted access to expensive drugs [83,84]. Inflammation is a fundamental and highly orchestrated physiological defensive process against noxious factors such as infections, exposure to toxicants, allergens, and other stimuli. Inflammation is often associated with pain that is quite often mediated with nonsteroidal drugs (NSAIDS) such as corticosteroids which possess remarkable anti-inflammatory activity and analgesics such as opioids and anticonvulsants [85,86]. However, the prolonged use of these drugs is discouraged due to their adverse effects such as severe gastric lesions, digestive system disorders, nausea, urinary retention, and dependence on opioids.

Six studies were included in this category from 2012 to 2020; five of them focused on *G. asiatica* whereas five articles focused on fruit parts and one study focused on the stem bark. The last study provided a comparison between the *n*-hexane extracts from *G. asiatica* and *G. optiva* for their protective effects against hypotonicity-induced lysis i.e., membrane stabilization. Lyophilization was the most commonly employed technique for sample preparation. The analgesic activity was evaluated using acetic acid-induced writhing and hot plate methods. Antipyretic activity was evaluated using the Brewerís yeast-induced pyrexia method, in vivo anti-inflammatory activity was recorded using carrageenan-induced paw edema, and in vitro anti-inflammatory activity was examined using the human RBC membrane stabilization method.

Four studies were included in the meta-analysis of the anti-inflammatory activities of two *Grewia* species i.e., *asiatica* and *optiva*, as summarized in Figure 5c, except one study reported by Bajpai et al. [61], where the results were not presented in any unit. The meta-analysis revealed that the *Grewia* species showed notable antinociceptive and anti-inflammatory activity (MRAW = 43.98, 95% CI = 21.98–65.97, *p* = 0.0, I^2^ = 100%) overall. However, a detailed sub meta-analysis suggested notable antinociceptive activities against acetic acid-induced writhing (MRAW = 72.18, 95% CI = 13.09–131.27, *p* = 0.0, I^2^ = 100%), anti-inflammatory activity against carrageenan-induced paw edema (MRAW = 45.18, 95% CI = 24.61–65.75, *p* = 0.0, I^2^ = 100%), and protection of the membrane against heat-induced hemolysis (MRAW = 21.6, 95% CI = −41.35–84.55, *p* = 0.0, I^2^ = 100%).

Das et al. [72] evaluated the anti-pain activity of aqueous extracts from *G. asiatica* fruits using the acetic acid-induced writhing (*n* = 35, trial duration 30 min), tail immersion (*n* = 35 trail duration was 10, 30, 60 min), and hot plate methods (*n* = 35, trial duration 10 min) in rats. Aqueous extracts of the *G. asiatica* fruit (200–300 mg/kg body weight) were found to attenuate the pain induced by acetic acid in the writhing test, tail immersion, and hot plate tests. Paviaya et al. [60] reported the analgesic efficacy of aqueous and methanolic extracts of *G. asiatica* bark in the hot plate test (*n* = 30, trial duration was 0, 30, 90, 190 min) and the writhing response test (*n* = 30, trial duration was 30 min). Similar studies also demonstrated that the methanolic and aqueous fruit extracts of *G. asiatica* at doses between 300 and 500 mg/kg counteracted the fever induced by lipopolysaccharide (*n* = 25, trail duration was 30, 60, 90 min) and brewer’s yeast (*n* = 25, trail duration was 1, 2, 3, 18 h) in rats, respectively [58,59]. The most recent study by Qamar et al. [24] found that 100% methanol and 50% aqueous methanol extracts of *G. asiatica* fruits protected the animals under experimentation from the painful stimulation of formalin (*n* = 40, trial duration was 0–25 min) in a dose-dependent manner with the maximum effect being 62.9% and 62.6%, respectively at 400 mg/kg/body weight. Similarly, methanol and aqueous methanol extracts of the *G. asiatica* fruit subjected to a glutamate-induced (*n* = 40, trial duration was 0–25 min) nociceptive response assessment in a mice model showed a significant anti-nociceptive effect from *G. asiatica* in comparison to the control and standard drug [24].

The anti-inflammatory potential of *G. asiatica* has also been extensively investigated. The data demonstrating the efficacy of the various anatomical fractions of *G. asiatica* such as bark as anti-inflammatory agents were significant when tested against carrageenan-induced paw oedema (*n* = 30 trail duration was 3 h) in rats. The authors confirmed that bark methanol and aqueous extracts were significant factors that attenuated paw edema at 400 mg/kg as 59.14% and 53.04%, respectively, while the response was quite comparable to that of indomethacin (64.02% reduction at 10 mg/kg) [60]. Methanol extracts of *G. asiatica* fruits were also screened for their possible anti-inflammatory activity on carrageenan-induced paw edema in rats at an oral dose level of 250 and 500 mg/kg, orally. The extract showed significant anti-inflammatory activity at both doses [61].

The methanol and aqueous extracts of *G. asiatica* fruits exerted anti-inflammatory activity against carrageenan-induced paw edema (*n* = 25, trail duration was 1–3 h) in a dose-dependent manner at 36.1% and 32.4% at 500 mg/kg, respectively in comparison to the standard indomethacin, which exerted 36.4% inhibition at 10 mg/kg [59]. Feeding 100% methanolic extracts of *G. asiatica* fruits to mice at the rate of 400 mg/kg b.w., inhibited formaldehyde (*n* = 40, trail duration was 0–25 h) and carrageenan-induced paw oedema (*n* = 40, trail duration was 1–3 h) by 74% and 71%, respectively, while the inhibition rate was 88% with indomethacin within 3 h of extract/standard drug feeding at 100 mg/kg. Further, a 50% methanolic extract also indicated increased efficacy against Prostaglandin E_2_ (PGE2)-induced paw edema (68.7% inhibition at 400 mg/kg (b.w.) in 120 min of extract administration) in comparison with the control while indomethacin presented a relatively higher rate of inhibition to PGE2-induced paw edema i.e., 79% at 100 mg/kg [24]. The last study reported that the traditional use of *G. asiatica n-*hexane extracts as anti-inflammatory ingredients was justified [62] on account of the extract’s ability to significantly stabilize human red blood cells in comparison with diclofenac potassium.

#### 3.2.4. Antidiabetic Activity

Type 2 diabetes has emerged as an important health problem within the 21st century [87]. Ever increasing infiltration trends of diabetes is one of the major health-threatening issues in both developed and developing societies and individuals [88]. Hitherto, one human, five animal, and three in vitro studies have been conducted to investigate the antidiabetic potential of *G. asiatica* from 2011 to 2016 and no other *Grewia* species have been explored under the mentioned category so far. Among nine reported studies, 3, 3, 1, 1 focused on leaves, fruits, bark, and pomace, respectively, and one study presented a comparison of the antidiabetic activity of the fruit, stem, and bark ethanolic extracts of *G. asiatica*.

Interestingly, the oral supplementation of an ethanol extract of the *G. asiatica* bark in alloxan-induced diabetic rats (*n* = 20, trial duration was 0–120 min) significantly attenuated the blood glucose levels and increased the survival rate of diabetic rats when compared with metformin-treated rats [89]. Likewise, ethanolic extracts of *G. asiatica* significantly lowered the blood glucose level in alloxan-induced diabetic rats (*n* = 36, trail duration was 0–7 h), and appeared to be more effective than glibenclamide used as a reference antidiabetic drug [70]. Another study on streptozotocin-induced diabetic rats (*n* = 36, trail duration was 0–24 h) recorded that the oral administration of extracts of the *G. asiatica* leaves at the rate of ~500 mg/kg b.w. for 21 days efficiently shortened and reduced blood glucose spikes in rats previously exposed to overloads of glucose, and considerably increased the glucose tolerance in normal rats [90].

Likewise, Khattab et al. [69] recorded normalized glycemia in streptozotocin-induced rats fed with *G. asiatica* fruit extracts. The study (*n* = 40, trail duration was 4 weeks) recorded reduced serum cytokine IL-1β and TNF-α levels, decreased pancreas malondialdehyde (MDA) levels, and normalized glycemia concomitantly with a higher accumulation of liver glycogen and increased liver and pancreas glutathione (GSH) and superoxide dismutase (SOD) enzyme activities. Moreover, the inhibitory properties of the aqueous extracts of the *G. asiatica* fruit against α-glucosidase and α-amylase activity with IC_50_ of 8.93 and 0.41 mg/mL, respectively were also reported by Das et al. [58] The inhibitory properties of the aqueous methanol extract of *G. asiatica* fruit residues with IC_50_ of 45.70 mg/mL against α-Amylase were recorded and were more promising when compared with the extracts of *B*. *vulgaris, A. comosus*, *A. lachoocha*, and *A. heterophyllus* fruit [30]. Notably, clinical trials revealed that *G. asiatica* fruit extracts have a moderate hypoglycemic effect on a non-diabetic human model. Furthermore, when tested in vitro with glucose, the fruit showed neutralizing effects on glucose-induced reactive oxygen species (ROS) suppression [73].

#### 3.2.5. Radioprotective and Hepatoprotective Potential

Exposure to ionizing radiations is unsafe for human health, even when used in therapeutics e.g., radiotherapy against cancer cells may cause severe side effects to irradiated patients.

Eight studies were reported in this category and all studies explored the edible portion of *G. asiatica*. Seven of them performed the extraction with methanol and one study employed ethanol extraction. Methanol extract supplementation was reported to protect mice brain lipids against radiation-induced (*n* = 120, trail duration was 1–30 days) oxidation and was shown to improve the GSH content by 14.3% [91]. *G. asiatica* fruit extracts were also shown to significantly protect against the deleterious effects of whole-body irradiation in mice [92]. Another study by Sisodia and Singh (2009) [93] reported that *G. asiatica* fruit extracts prevented radiation-induced memory and learning deficits in addition to known histopathological, biochemical, and behavioral ameliorative effects. Numerous studies advocated a *G. asiatica* fruit-enriched diet to reduce lipid peroxidation rates and serum cholesterol, and to restore the normal levels of GSH, glutathione peroxide (GSH-Px), sugars, and proteins in irradiated mice models [74,94,95]. A histopathological and biochemical investigation of the hepatic tissues of X-ray-irradiated *G. asiatica* extract-fed mice demonstrated hepatoprotective effects [2,96]. Radioprotective effects were also noticed in histopathological specimens of mice testis where irradiation resulted in lower spermatogonia “A”, spermatogonia “B”, spermatocytes and spermatid count when compared with animals irradiated after supplementation with *G. asiatica* fruit extracts [75].

#### 3.2.6. Antimicrobial Properties

Plants serve as an important source of novel medicinal substances [76,97]. Sufficient information is available to confirm the anti-infective role of bioactive compounds of natural origin. For centuries, the use of herbal drugs has been extensively recommended to modulate various opportunistic infections. Flavonoids isolated from ethnopharmacologically established plants are considered to be effective antimicrobial substances against a wide variety of microorganisms [77,98]. Nine studies were reported in the mentioned category from 2011 to 2020, wherein two studies performed both antibacterial and antifungal activities, five studies reported only antibacterial activities, and lastly, two studies only evaluated the antifungal potential. Out of nine total reported articles, *G. asiatica* was the most commonly explored i.e., *G. asiatica* was the focus in six studies and *G. optiva*, *G. lasiocarpa,* and *G. hirsuta* were the focus in 1, 1, and 1 studies, separately. Five studies focused on leaves, two studies focused on fruit, and two studies used stem bark to evaluate the antibacterial and antifungal properties of the *Grewia* species. Researchers have shown that crude extracts of the *Grewia* spp. have valuable antibacterial activities predominately associated with their high flavonoid content. Beside the fruit fraction, the leaves and stem bark of *G. asiatica* have also been suggested to possess antimicrobial potential [27,63,64,65,66,68].

Six studies were included in the meta-analysis of antimicrobial potential including three *Grewia* species i.e., *asiatica*, *optiva*, and *hirsuta* as summarized in Figure 5d, except three studies reported by Akwu et al. (2020) [15], Zia et al. (2011) [63], Dawar et al. (2020) [68] wherein standard deviation was not mentioned. The meta-analysis revealed that the *Grewia* species showed notable antibacterial and anti-fungal activity (MRAW = 17.15, 95% CI = 11.59–22.70, *p* = 0.0, I^2^ = 100%) overall. However, the detailed sub meta-analysis suggested showed notable antibacterial (MRAW = 10.05, 95% CI = 7.72–12.38, *p* = 0.0, I^2^ = 100%) and anti-fungal (MRAW = 32.51, 95% CI = 26.85–38.17, *p* = 0.0, I^2^ = 100%) activities.

Flavonoids and flavonoid-rich fractions isolated from the peel and pulp of *G. asiatica* caused significant inhibition against Gram-positive and Gram-negative bacterial strains. *Staphylococcus aureus* was reported to be the most susceptible and *Bacillus subtilis* was reported to be the least susceptible among the Gram-positive bacterial strains while *Salmonella typhi* were the most susceptible and *Escherichia coli* ranked among least susceptible among the Gram-negative bacterial strains [64].

The antibacterial activity of the methanolic extract of the *G. asiatica* leaves has been reported against *Staphylococcus aureus* and *Salmonella typhi* while the aqueous extract of *G. asiatica* leaves was only found to be effective against *S. aureus* [66]. *G. asiatica* fruit extracts can inhibit Gram-negative bacteria through their bioactive compounds such as flavonoids, alkaloids, and saponins without necessarily penetrating into the microbial cell [99]. The potent antibacterial activity of *G. asiatica* leaf extracts has also been shown against eight different bacterial strains, i.e., *Proteus mirabilis*, *Citrobacter* sp., *Pseudomonas aeruginosa*, *Escherichia coli*, *Salmonella typhi*, *Micrococcus luteus*, *Staphylococcus aureus*, and *Bacillus subtilis* [63]. Another study investigating the efficacy of the bark and fruit extracts of *G. asiatica* against four Gram-positive and six Gram-negative bacterial strains found that the extracts were more active toward *S. aureus*, *E. coli*, and *Proteus vulgaris*, and overall, were more active on Gram-positive strains as compared to Gram-negative bacteria [65]. Another study found the aqueous extract of *G. optiva* leaves to exert moderate inhibition against three different bacterial strains named *S. aureus*, *E. coli*, *Salmonella typhi*, and *Streptococcus pneumoniae* [27]. A seventy per cent methanol extract of *G. hirsuta* showed antibacterial activity against *S. aureus* and *E. coli* equivalent to the standard drug ciprofloxacin [67].

Ethanol extracts of *G. asiatica* leaves were reported to have good antifungal activity against nine fungal strains, namely, *Aspergillus effusus*, *A. parasiticus*, *A. niger*, *Saccharomyces cerevisiae*, *Candida albicans*, *Yersinia aldovae*, *Fusarium solani*, *Macrophomina phaseolina*, and *Trichophyton rubrum* [63]. Pathogenic fungi are responsible for huge crop production losses by perishing the roots system within plants. In vitro antifungal trials using paper disc and diffusion methods found that a 100% aqueous extract from the *G. asiatica* leaves induced significant inhibition against *Rhizoctonia solani*, *Fusarium oxysporum*, and *Macrophomina phaseolina* and consequently ameliorated the growth of bottle gourd and cowpea. Correspondingly, in vivo results disclosed that an addition of 1% of the powder of the *G. asiatica* leaves to organic matter considerably reduced the colonization of *Macrophomina phaseolina, Rhizoctonia solani*, and *Fusarium spp*. An even greater inhibition against colonization was offered by a 100% *G. asiatica* leaf extract when directly drenched into the soil. Further, seed treatment of food crops with a 100% leaf extract was reported to increase the bottle gourd and cowpea growth and notably suppressed fungal attack [68].

In an in vitro antiviral trial, a *G. asiatica* extract was sprayed on test plants at different concentrations (500, 1000, 1500, and 2000 μg/mL) against ULCV (Urdbean leaf crinkle virus). Plants sprayed with 1000 μg/mL of a *G. asiatica* extract exhibited a minimum % infection (34%) as compared to the control which showed 90% infection, while notable activity against ULCV at concentrations of 1500 and 2000 μg/mL was observed [100]. The traditional use of the *G. asiatica* fruit and its decoctions as a remedy for digestive and urinary disorders is hence justifiably linked to the broad-spectrum antimicrobial activity of the fruit extracts against digestive and urinary tract pathogens such as *Salmonella*, *E. coli*, *S. aureus*, *P. aeruginosa*, and *M. luteus.* In a recent study by Goswami et al. (2018) [1], it was reported that a *G. asiatica* leaf acetone extract inhibited the activity of different pathogenic fungi including *A. fumigatus* and *C. glabratai* at a concentration of 35 mg/mL.

#### 3.2.7. Antiemetic and Antimalarial Activities

The antiemetic potential of an ethanol extract of the *G. asiatica* fruit was evaluated in a canine model at quite low doses while acute oral toxicity assays proved that extracts were safe for consumption at 200 mg/kg b.w., [101]. The referred study documented that administration of fruit extracts at 120 mg/kg b.w., was capable of inducing antiemetic effects in dogs and standard antiemetic drugs such as largactil and maxolon were shown to be active. Similar assays were performed on male chicks and the researchers suggested a dose-dependent inhibition, i.e., a decrease in the number of retches such that 39% inhibition was observed at 50 mg/kg while ~60% inhibition was recorded with a 100 mg/kg supplementation of methanol fruit extracts of *G. asiatica* [71]. The literature confirmed the anti-malarial potential (69% inhibition) of the *G. asiatica* leaves assayed for their possible anti-malarial activity using Enoyl-ACP reductase inhibitory assay [102].

#### 3.2.8. Other Activities: Immunomodulatory, Anti-Depressant, Anti-Neurodegenerative, Drug Delivery Polymers

The clinical data are still scarce on the immunomodulatory or immunoregulatory properties of *G. asiatica.* However, the presence of bioactive compounds bearing significant immune-mediating activities hints at future trends in immunological research related to the genus *Grewia*. As discussed earlier, the fruit extracts of *G. asiatica* carry a significant concentration of compounds such as quercetin, isovitexin, kaempferol, iso-liquiritigenin, and umbelliferone that have been extensively explored for their innate and adoptive immune response in inflammatory disorders [103,104,105,106]

A flavonoid-rich ethanol extract of the *G. asiatica* leaves was reported to exhibit immunomodulatory properties with satisfactory immunostimulation [10]. The notable sedative–hypnotic potential of methanol leaf extracts of *G. asiatica* in mice models was investigated and no toxicity was observed at a 300 mg/kg dose level [107]. Further studies explicated that *G*. *asiatica* methanol extracts improved scopolamine-induced learning and memory deficits in rats through the employment of behavior assessment models by reinstating the cytoarchitecture of effected neuronal cells, elevating neurotransmitter acetylcholine, and settling oxidative stress [11]. *G. asiatica* leaf fractions derived using petroleum ether and chloroform solvents showed considerable effectiveness against neurodegenerative ailments by inhibiting bovine brain acetylcholinesterase (IC_50_ = 55.88 µg/mL) and human blood butyrylcholinesterase (IC_50_ = 26.14 µg/mL) enzymes, respectively [12]. *G. asiatica* extracts have the ability to generate colloidal dispersions and viscous gel in water. The mucilage of *G. asiatica* was therefore tested as natural polymeric ingredients for gel formulation in drug design and suggested identical behavior to that of marketed formulation without negatively affecting drug release [108].

## 4. Discussion

From the results of the selected papers on the nutritional, phytochemical composition, and health-promoting potential of the *Grewia* species, our review identified primary metabolites (carbohydrate, protein and amino acids, fiber, fat, and fatty acids), minerals (calcium, sodium, iron, zinc, manganese), vitamins, and phytochemicals including flavonoids (flavones and anthocyanidins), phenolic acids, and triterpenes as major classes. These findings underscore the importance of this genus in maintaining a healthy and balanced diet. In comparison to the fruits, we discovered that the leaves and seeds have a better nutritional value and a larger quantity of bioactive substances. Although the composition varies according to the *Grewia* species, in general, the *Grewia* species are high in protein and fiber and have a low to intermediate fat and carbohydrate content, making them an excellent choice for people who are trying to lose weight.

Importantly, the contents of minerals such as calcium, potassium, sodium, iron, zinc, and manganese were found in notable amounts. The Institute of Medicine [109] recommended a daily allowance (RDA) of calcium as 1000 mg/day for adults (19–50 years) wherein 100 g of the powder of the *G. asiatica* seed, *G. tenax* fruit, and *G. villosa* fruit can cover approximately 82%, 78%, and 54%, respectively of the RDA for calcium i.e., important for bone health. In the same manner, 100 g of the powder of *G. asiatica* seeds, *G. villosa* fruits, *G. flavescence* fruits, and *G. tenax* fruits can cover 100% of the RDA for iron. The Institute of Medicine [109] suggested 8 mg/day iron for all age groups of men and postmenopausal women. That functions as a component of a number of proteins, including enzymes and hemoglobin, the latter being important for the transport of oxygen to tissues throughout the body for metabolism [110]. A hundred grams of the fruit powder of *G. tenax, G. flavescence,* and *G. villosa* can fulfil the RDA of zinc up to 23%, 13%, and 19% as the zinc RDA for adults is 8 mg/day for women and 11 mg/day for men [109]. Zinc functions as a component of various enzymes in the maintenance of the structural integrity of proteins and in the regulation of gene expression [110]. The RDA for manganese is 2.3 and 1.8 mg/day, respectively for adult men and women [109] and 100 g of *G. tenax* fruit powder can cover 100% RDA of manganese whereas the powder of *G. asiatica* fruits and seeds can satisfy almost 50% of the RDA of manganese, which is involved in the formation of bones and in the amino acid, lipid, and carbohydrate metabolisms [109,110]. The RDA of ascorbic acid is 90 mg/day as per the guidelines of the Food and Nutrition Board [111] and 100 g of the powder of the *G. asiatica* seeds can fulfill 5.7% of the RDA of vitamin C which is involved in the maintenance of normal connective tissue, wound healing and is needed for bone remodeling. It also acts as an antioxidant, opposes mutation in DNA, and is utilized in the treatment of several cancers [112]. A hundred grams of the fruit powder of *G. tenax*, *G. flavescence,* and *G. villosa* can contribute towards the adequate intake (AI) of potassium at 16.3%,17.5%, and 19.6%, respectively, and the Food and Nutrition Board [111] suggested that the AI of potassium should be up to 4700 mg/day. Potassium is responsible for acid-base control, maintaining osmotic pressure, nerve impulse transmission, muscular contraction, and the transport of carbon dioxide and oxygen [113,114]. The fatty acid profiling of two *Grewia* species i.e., *G. asiatica* and *G. bicolor* suggested the presence of saturated and unsaturated fatty acids. The polyunsaturated fatty acids are dominant in concentration as compared to saturated fatty acids. Polyunsaturated fatty acids in the diet should be increased since they contribute to lower total plasma cholesterol and protect against cardiovascular disease [115]. The intake of saturated fatty acids is linked to hypercholesterolemia and heart problems [116]. Both species had a moderate amount of saturated fatty acids such as palmitic acid (12.17–11.46%) and stearic acid (5.01–5.77%). Stearic acid has been demonstrated to worsen coronary artery disease by reducing high density lipoprotein cholesterol [116,117,118]. Studies have shown that palmitic acid is a potent inducer of DNA damage in insulin-secreting cell linen [119]. In contrast, unsaturated fatty acids including oleic acid and linoleic acid are reported in notable amounts in *G. asiatica* and *G. bicolor* seed oils ranging between 16.31–19.33% and 60.06%, 53.21%, respectively. Oleic acid is an ω-9 unsaturated fatty acid known to improve high-density lipoprotein (HDL) cholesterol while lowering low-density lipoprotein (LDL) cholesterol, lowering the risk of heart disease and atherosclerosis [120]. Oleic acid also prevents breast cancer cells from proliferating by inhibiting the growth of cancer-causing oncogenes HER-2/neu (erbB-2) expression [121]. Diets high in oleic acid have been demonstrated to decrease slightly obese women lose weight [122].

The phytochemicals identified in this review establish linkages to the underlying mechanisms on the health benefits of *Grewia* species. Most of the *Grewia* species compounds are known to have several health benefits, including antioxidant, anti-inflammatory, anticancer, hepatoprotective, radioprotective, and antimicrobial aspects. The antioxidant activity of the *Grewia* species lies mainly in its leaves, seeds, and pulp since they possess a higher radical scavenging ability while peel and stem bark extracts possess non-influential activity, confirmed in in vitro research mediated by their higher content on flavonoids, phenolic acids, and triterpenes. Regarding the anti-inflammatory properties, the fruit, bark, and leaf extracts of *Grewia* species and mainly the *asiatica* species controlled the pain mediation by suppressing the pro-inflammatory cytokines during in vivo assays and also imparted protection to red blood cell membrane against heat-induced hemolysis. In the anticancer analysis, fruit extracts exhibited remarkable activity followed by the leaf extracts, but the fruit residues and the stem bark extracts showed non-influential activity that is consistent with their bioactive metabolite potential. Studies have shown an improvement of glycemic profile by reducing serum glucose level, inhibiting α-amylase, and α-glucosidase evaluated using in vitro and in vivo research. The *Grewia* species may also facilitate antimicrobial activity associated with an inhibitory effect on Gram-positive and negative bacteria’s growth and acting as an antifungal.

Quercetin, chlorogenic acid, caffeic acid, morin, and catechin were the compounds identified in more than one paper. A plethora of literature is available on the biological activities of the listed compounds from different plant sources. The literature cited below correlates the biological activity of the key compounds reported in this study with the existing set of information wherein these compounds have been individually explored for their antioxidant, anti-inflammatory, anticancer, and antimicrobial properties.

Quercetin retrieved from the methanol extract of *Asparagus cochinchinensis* had notable antioxidant activity with an IC_50_ of 14.52 µg/mL against free radicals, i.e., DPPH in contrast to standard vitamin C recorded with an IC_50_ of 10.49 µg/mL [123]. Notable free radical scavenging activity i.e., 6.35 μM (SC_50_) was reported for chlorogenic acid isolated from the *n*-butanol fraction of the *Eriobotrya japonica* leaves [124]. Caffeic acid was reported to inhibit the DPPH free radicals with an EC_50_ of 111 mg/mL [125]. The compound was also found to be effective at reducing ferric iron with a FRAP value of 11.50 μmol Fe(II)/g d.w. Flavonoids (i.e., catechin, morin) and phenolic acids (i.e., caffeic acid, chlorogenic acid) were reported to exhibit notable antioxidant potential in four different biological assays including ORAC, FRAP, ABTS and DPPH [126]. Regarding the anti-inflammatory activity of the key identified compounds from various *Grewia* species, the intraperitoneal administration of quercetin at 80 mg/kg was reported to alter the carrageenan-induced paw edema in rats [127]. Methanolic extracts of *Cheilanthes farinosa* are potential carriers of chlorogenic acid. In a study by Yonathan et al. [128], the authors suggested that 10 mg/mL of chlorogenic acid had a remarkable anti-inflammatory activity against edema comparable with that of acetyl salicylic acid at a relatively higher concentration i.e., 200 mg/mL. Inhibition of inflammation in carrageenan-induced paw edema using catechin in mice was reported to be approximately 28% at a dose of 30 mg/kg [129].

Plausible information on the anticancer activities of plants derived from bioactive compounds including flavonoids exists. This section briefly describes the findings of studies on the anticancer properties of plants originating from bioactive compounds. Quercetin isolated from the methanol extract of *Asparagus cochinchinensis* was reported to exhibit strong cytotoxicity against the HeLa cell line (IC_50_ of 5.78 µg/mL), followed by NCI–H460 (IC_50_ of 12.57 µg/mL), Hep-G2 (IC_50_ of 20.58 µg/mL), and MCF-7 with an IC_50_ of 31.04 µg/mL [123]. Catechin isolated from green tea has been reported to inhibit the proliferation of lung cancer cells through the upregulation of the let-7 signaling pathway and the downregulation of the C-MYC, LIN-28 signaling pathway [130]. Previously, some reports have suggested that morin showed a diverse range of biological functions and has been reported to play essential roles in suppressing the growth of cancer cells (HepG2, HT29, and HCT116) as shown by Hussain et al. [131]. Morin-treated lung (A549) cells showed a decreased cell viability, colony formation, and migration rate when compared with the dimethyl sulfoxide-treated cells by suppressing the expression of miR-135b [132]. Chlorogenic acid derived from *Laurocerasus officinalis* was reported to exert notable inhibition against the breast cancer cell line (MCF-7) with IC_50_ 30.9 μg/mL [133]. Chlorogenic acid has been reported to inhibit the proliferation of human lung cancer (A549) cell lines by targeting annexin A2 in vitro and in vivo [134]. Chlorogenic acid and caffeic acid exhibited strong cytotoxic activity in vitro against A549 lung cancer cells, with an IC_50_ value of 9.8 μM and 8.9 μM, which was similar to that of the positive control 5-fluorouracil i.e., 3.8 μM [135]. In an earlier study by Garcia et al. [136], caffeic acid isolated from *Scrophularia frutescens* was reported to exhibit ID_50_ of 28.55 × 10^−3^ μM against the Hep-2 cell line i.e., derived from a human epidermoid carcinoma of the larynx. A total of 10 μM quercetin was recorded to reduce the expression of the immunoreactive P-glycoproteins (Pgp) in MCF-7 ADR-resistant cells. Myricetin was also reported to suppress breast cancer metastasis through down-regulating the activity of the metalloproteinase matrix (MMP)-2/9 [137]. Another study by Rajendran et al. [138] reported that plant-based myricetin exhibited cytotoxic potential by inducing cell cycle arrest and ROS-reliant mitochondria-facilitated apoptosis in A549 lung cancer cells.

Regarding the antimicrobial activity, quercetin was reported to inhibit *S. aureus* and *P. aeruginosa* at dose of 20 mg/mL while *P. vulgaris* and *E. coli* were inhibited at a concentration 300 mg/mL and 400 mg/mL, respectively [139]. Recently, a combined treatment of caffeic acid and UV-A LEDs effectively inactivated *E. coli*, *S. Typhimurium*, and *L. monocytogenes* in both a phosphate buffered saline (PBS) and commercial apple juice with no adverse effect on quality [140]. The antibacterial activity of morin was tested against three bacterial strains named *E. coli*, *K. pneumoniae*, *S. aureus* wherein at a concentration of 100 µg/cylinder morin was effectively inhibited all strains [141].

## 5. Conclusions

The *Grewia* species contains biologically significant amounts of primary metabolites such as carbohydrates, protein and amino acids, ash and minerals, and fiber, but low contents of fats and fatty acids. These characteristics make them a good choice for a healthy life and for weight conscious people. Other than that, crude extracts of various parts i.e., the fruit, stem, bark, leaves, seeds, and identified/quantified compounds, including gallic acid, chlorogenic acid, caffeic acid, quercetin, morin, myricetin, vitexin, and catechins can be used for the development of nutraceuticals in order to address life-threatening ailments.

The present review discussed in detail the health-promoting potential of the various anatomical parts of all included *Grewia* species and the compounds extractable from those parts. Future studies should be conducted to isolate the identified compounds from *G. asiatica* and to conduct their clinical investigations and safety assessment. We also encourage researchers to work on other *Grewia* species for nutritional and phytochemical profiling so a comparison can be drawn, enabling an identification of the “best” species, from a bioactive and therapeutics point of view. A bibliometric analysis of co-authorships highlighted that most of the authors and regions of study are from South Asia, mainly India and Pakistan. So far, authors from India have collaborated and explored the antioxidant, anti-inflammatory, anticancer, radioprotective, and hepatoprotective aspects of the *Grewia* species, whereas authors from Pakistan have collaborated and evaluated antioxidant, anti-inflammatory, anticancer, antibacterial, antiemetic, and antimalarial activities. Surprisingly, the antibacterial, and antimalarial aspects were not explored by Indian authors, and the antidiabetic, hepatoprotective, and radioprotective potential were not explored by Pakistani authors as of the date of the present review. The mentioned loop could motivate the authors from other geographical regions, where the *Grewia* species also grows, to join the international ethno-geo-pharmacological investigation and to provide a comprehensive evaluation of the bio-potency by applying a unified methodology. Providing a unified specification of this potential *Grewia* genus and its parts (seeds, stems, roots, leaves and fruits) and identified compounds (quercetin, myricetin, morin, catechins, gallic acid, chlorogenic acid, caffeic acid, and others) would allow researchers to make a geo-biopotency relationship based on plant growth in the different regions, exhibiting various high altitudes, sun exposure time, climate, soil type, humidity, and irrigation methods.

## Figures and Tables

**Table 2:** In vitro and in vivo biological activities of the *Grewia* species.

Antioxidant Effects of *Grewia species*											
*Species*	Plant Part	Origin	Extraction Solvent	Activity	Assay	Activity of Extract	Std Dev	Positive Control	Activity	Std Dev	References
*G. asiatica*	Leaf	India	Acetone	Antioxidant	DPPH	127.5 IC_50_ µg/mL	0.8	NG	NG	NG	[1]
*G. asiatica*	Fruit	Pakistan	50% aqueous Methanol	Antioxidant	DPPH	41 IC_50_ µg/mL	0.1	Ascorbic acid	75.1 IC_50_ µg/mL	0.01	[24]
*G. asiatica*	Fruit	Pakistan	Methanol	Antioxidant	DPPH	77 IC_50_ µg/mL	1.1	Ascorbic acid	75.1% inhibition	0.01	[24]
*G. asiatica*	Leaf	India	Benzene	Antioxidant	DPPH	16.19 IC_50_ µg/mL	2.1	Ascorbic acid	78.1 IC_50_ µg/mL	4.05	[46]
*G. optiva*	Leaf	Pakistan	Water	Antioxidant	DPPH	60 IC_50_ µg/mL	0.6	Ascorbic acid	28 IC_50_ µg/mL	0.40	[27]
*G. lasiocarpa*	Stem	South Africa	Chloroform	Antioxidant	DPPH	>1000 IC_50_ µg/mL	0.2	NG	NG	NG	[15]
*G. asiatica*	Fruit	Pakistan	80% aqueous Methanol	Antioxidant	DPPH	85% inhibition	1.5	BHA	89% inhibition	NG	[48]
*G. asiatica*	Fruit	India	Methanol	Antioxidant	DPPH	84.8% inhibition	0.9	NG	NG	NG	[49]
*G. asiatica*	Fruit	Pakistan	Methanol	Antioxidant	DPPH	>60% inhibition	0.7	NG	NG	NG	[50]
*G. flava*	Peel	Botswana	Ethanol	Antioxidant	DPPH	375 µmol GAE/g	1.1	NG	NG	NG	[52]
*G. biocolor*	Peel	Botswana	Ethanol	Antioxidant	DPPH	165 µmol GAE/g	0.2	NG	NG	NG	[52]
*G. asiatica*	Leaf	India	Water	Antioxidant	NO	1098 IC_50_ µg/mL	0.9	NG	NG	NG	[51]
*G. asiatica*	Leaf	India	Benzene	Antioxidant	NO	27.0 IC_50_ µg/mL	1.6	Ascorbic acid	20.5 IC_50_ µg/mL	1.7	[46]
*G. asiatica*	Seed	Pakistan	Ethyl acetate	Antioxidant	ABTS	55.8 TEAC µmol/g	0.3	NG	NG	NG	[47]
*G. asiatica*	Peel	Pakistan	70% aqueous acetone	Antioxidant	ABTS	107.2 TEAC µmol/g	2.4	NG	NG	NG	[47]
*G. asiatica*	Pulp	Pakistan	70% aqueous acetone	Antioxidant	ABTS	60.9 TEAC µmol/g	1.8	NG	NG	NG	[47]
*G. asiatica*	Fruit	Pakistan	Methanol	Antioxidant	ABTS	% inhibition >60%	0.7	NG	NG	NG	[50]
*G. optiva*	Leaves	Pakistan	Water	Antioxidant	ABTS	70 IC_50_ µg/ml	0.8	Ascorbic acid	30 IC_50_ µg/mL	0.30	[27]
*G. asiatica*	Fruit	India	Methanol	Antioxidant	FRAP	4.14 mg GAE/g	1.1	NG	NG	NG	[49]
*G. asiatica*	Fruit	Pakistan	50% Aqueous methanol	Antioxidant	FRAP	43 mg GAE/g	0.6	Ascorbic acid	15.0 mg GAE/g	0.01	[24]
*G. asiatica*	Fruit	Pakistan	Methanol	Antioxidant	FRAP	27 mg GAE/g	0.7	Ascorbic acid	15.0 mg GAE/g	0.01	[24]
*G. lasiocarpa*	Stem	South Africa	Chloroform	Antioxidant	FRAP	>1000 IC_50_ µg/mL	0.9	NG	NG	NG	[15]
*G. asiatica*	Fruit	Pakistan	50% Aqueous methanol	Antioxidant	H_2_O_2_	73% inhibition	0.5	Ascorbic acid	79.1% inhibition	0.02	[24]
*G. asiatica*	Fruit	Pakistan	Methanol	Antioxidant	H_2_O_2_	43% inhibition	0.4	Ascorbic acid	79.1% inhibition	0.02	[24]
Anticancer effects of *Grewia species*											
***Species***	**Plant Part**	**Origin**	**Extracting Solvent**	**Activity**	**Assays**	**Cancer Cell Line**	**Activity of Extract** **(IC_50_ )**	**Reference Drug**	**Activity** **(IC_50_)**	**Std Dev**	**References**
*G. asiatica*	Fruit	India	Water	Anticancer	MTT	HEp-2	50.31 µg/mL	Methotrexate	0.98 µg/mL	NG	[55]
*G. asiatica*	Leaves	India	Aqueous	Anticancer	MTT	HEp-2	61.23 µg/mL	Methotrexate	0.98 µg/mL	NG	[55]
*G. asiatica*	Fruit residue	India	Methanol	Anticancer	MTT	HEp-2	>250 µg/mL	Not given	NG	NG	[30]
*G. asiatica*	Fruit	Pakistan	Aqueous methanol	Anticancer	MTT	HEp-2	80.41 µg/mL	Methotrexate	0.82 µg/mL	NG	[24]
*G. asiatica*	Fruit	Pakistan	Aqueous methanol	Anticancer	MTT	HEp-2	80.41 µg/mL	Methotrexate	0.82 µg/mL	NG	[24]
*G. asiatica*	Fruit	India	Aqueous	Anticancer	MTT	NCI-H522	59.03 µg/mL	Methotrexate	0.96 µg/mL	NG	[55]
*G. asiatica*	Leaves	India	Methanol	Anticancer	MTT	NCI-H522	Notable cytotoxicity	NG	NG	NG	[56]
*G. asiatica*	Fruit	Pakistan	Aqueous methanol	Anticancer	MTT	NCI-H522	73.01 µg/mL	Methotrexate	0.91 µg/mL	0.21	[24]
*G. asiatica*	Fruit	Pakistan	Aqueous methanol	Anticancer	MTT	NCI-H522	73.01 µg/mL	Methotrexate	0.91 µg/mL	0.21	[24]
*G. asiatica*	Fruit	India	Aqueous	Anticancer	MTT	MCF-7	58.65 µg/mL	Methotrexate	0.98 µg/mL	0.4	[55]
*G. asiatica*	Leaves	India	Aqueous	Anticancer	MTT	MCF-7	50.37 µg/mL	Methotrexate	0.98 µg/mL	0.4	[55]
*G. asiatica*	Leaves	India	Methanol	Anticancer	MTT	MCF-7	Notable cytotoxicity	NG	NG	NG	[56]
*G. asiatica*	Leaves	India	Methanol	Anticancer	MTT	MCF-7	199.5 µg/mL	NG	NG	NG	[57]
*G. asiatica*	Fruit residue	India	Methanol	Anticancer	MTT	MCF-7	68.91 µg/mL	NG	NG	NG	[30]
*G. asiatica*	Fruit	Pakistan	Aqueous methanol	Anticancer	MTT	MCF-7	34.87 µg/mL	Methotrexate	0.82 µg/mL	0.1	[24]
*G. asiatica*	Fruit	Pakistan	Aqueous methanol	Anticancer	MTT	MCF-7	34.87 µg/mL	Methotrexate	0.82 µg/mL	0.1	[24]
*G. lasiocarpa*	Stem bark	South Africa	Chloroform	Anticancer	MTT	MCF-7	>1000 µg/mL	NG	NG	NG	[15]
*G. asiatica*	Leaves	India	Methanol	Anticancer	MTT	Hela	177.8 µg/mL	NG	NG	NG	[57]
*G. asiatica*	Fruit residue	India	Methanol	Anticancer	MTT	Hela	>100 µg/mL	NG	NG	NG	[30]
*G. lasiocarpa*	Stem bark	South Africa	Chloroform	Anticancer	MTT	Hela	>1000 µg/mL	NG	NG	NG	[15]
*G. asiatica*	Fruits	Pakistan	Methanol	Anticancer	MTT	Hela	406.5 µg/mL	Methotrexate	0.89	0.31	[24]
*G. asiatica*	Fruits	Pakistan	Aqueous methanol	Anticancer	MTT	Hela	282.4 µg/mL	Methotrexate	0.89	0.31	[24]
*G. asiatica*	Leaves	India	Methanol	Anticancer	MTT	K-562	54.90 µg/mL	NG	NG	NG	[57]
*G. asiatica*	Leaves	India	Methanol	Anticancer	MTT	HL-60	53.70 µg/mL	NG	NG	NG	[57]
*G. lasiocarpa*	Stem bark	South Africa	Chloroform	Anticancer	MTT	HEK293	No Activity	NG	NG	NG	[15]
Anti-inflammatory properties of *Grewia species*											
***Species***	**Plant Part**	**Origin**	**Extracting Solvent**	**Activity**	**Assay**	**Negative Control** **(% Inhibition)**	**Activity of Extract** **(% Inhibition)**	**Std Dev**	**Positive Control (% Inhibition)**	**Std Dev**	**References**
*G. asiatica*	Fruit	India	Water	Analgesic	Acetic acid induced writhing	none	99.39 at 300 mg/kg	0.21	99.18 at 400 mg/kg of aspirin	0.4	[58]
*G. asiatica*	Fruit	Pakistan	Methanol	Analgesic	Acetic acid induced writhing	none	% inhibition was 61.81 at 500 mg/kg	0.54	75.1 at 10 mg/kg of indomethacin	0.89	[59]
*G. asiatica*	Fruit	Pakistan	Water	Analgesic	Acetic acid induced writhing	none	% inhibition was 55.34 at 500 mg/kg	0.34	75.1 at 10 mg/kg of indomethacin	0.89	[59]
*G. asiatica*	Fruit	India	Water	Antipyretic	Hot plate method	Hot plate reaction time was 3.1 min	Hot plate reaction time was 7.4 min at 400 mg/kg	1.07	Hot plate reaction time was 2.12 min at 300 mg/kg of Aspirin	0.42	[58]
*G. asiatica*	Bark	India	Methanol	Analgesic	Hot plate method	Hot plate reaction time was 2.80 sec	Hot plate reaction time was 12.37 sec at 400 mg/kg	1.42	Hot plate reaction time was 13 sec at 300 mg/kg at 5 mg/kg of Pentazocine	0.84	[60]
*G. asiatica*	Bark	India	Methanol	Analgesic	Hot plate method	Hot plate reaction time was 2.80 sec	Hot plate reaction time was 12 sec at 400 mg/kg	1.38	Hot plate reaction time was 13 sec at 300 mg/kg at 5 mg/kg of Pentazocine	0.84	[60]
*G. asiatica*	Fruit	Pakistan	Methanol	Antipyretic	Breweris yeast induced pyrexia	Average temperature was 102	Average temperature was 100.81 at 500 mg/kg	0.19	Average temperature was 98.6 at 150 mg/kg of paracetamol	0.04	[59]
*G. asiatica*	Fruit	Pakistan	Water	Antipyretic	Brewerís yeast induced pyrexia	Average temperature was 102	Average temperature was 100.5 °C at 500 mg/kg	0.12	Average temperature was 98.6 °C at 150 mg/kg of paracetamol	0.04	[59]
*G. asiatica*	Bark	India	Methanol	Anti-inflammatory	Carrageenan- induced paw oedema	% inhibition was 0	% inhibition was 59.14 at 400 mg/kg	0.51	% inhibition was 64.2 at 10 mg/kg of indomethacin	0.38	[60]
*G. asiatica*	Bark	India	Water	Anti-inflammatory	Carrageenan- induced paw oedema	% inhibition was 0	% inhibition was 53.04 at 400 mg/kg	0.39	% inhibition was 64.2 at 10 mg/kg of indomethacin	0.38	[60]
*G. asiatica*	Fruit	India	Methanol	Anti-inflammatory	Carrageenan- induced paw oedema						[61]
*G. asiatica*	Fruit	Pakistan	Methanol	Anti-inflammatory	Carrageenan- induced paw oedema	% inhibition was 0	% inhibition was 36.12 at 500 mg/kg	0.43	% inhibition was 36.4 at 10 mg/kg of indomethacin	0.03	[59]
*G. asiatica*	Fruit	Pakistan	Water	Anti-inflammatory	Carrageenan- induced paw oedema	% inhibition was 0	% inhibition was 32.44 at 500 mg/kg	0.21	% inhibition was 36.4 at 10 mg/kg of indomethacin	0.03	[59]
*G. asiatica*	Leaves	India	*n*-Hexane	Anti-inflammatory	Membrane protection	% inhibition was 0	% inhibition was 80.91 at 600 µg/mL	NG	% inhibition was 21.1 at 600 µg/mL at 600 µg/mL of diclofenac potassium	NG	[62]
*G. asiatica*	Leaves	India	Methanol	Anti-inflammatory	Membrane protection	% inhibition was 0	% inhibition was 2.5 at 600 µg/mL	NG	% inhibition was 21.1 at 600 µg/mL at 600 µg/mL of diclofenac potassium	NG	[62]
*G. optiva*	Leaves	India	*n*-Hexane	Anti-inflammatory	Membrane protection	% inhibition was 0	% inhibition was 0 at 600 µg/mL	NG	% inhibition was 21.1 at 600 µg/mL at 600 µg/mL of diclofenac potassium	NG	[62]
*G. optiva*	Leaves	India	Methanol	Anti-inflammatory	Membrane protection	% inhibition was 0	% inhibition was 3.00 at 600 µg/mL	NG	% inhibition was 21.1 at 600 µg/mL at 600 µg/mL of diclofenac potassium	NG	[62]
Antimicrobial activities of *Grewia species*											
***Species***	**Plant Part**	**origin**	**Extracting Solvent**	**Activity**	**Bacterial/Fungal Strain**	**Activity of Extract**	**Std Dev**	**Positive Control**	**Activity**	**Std Dev**	**References**
*G. asiatica*	Leaves	Pakistan	Ethanol	Antibacterial	*S. aureus*	MIC was >1 mg/mL	NG	Amoxicillin	MIC was 20 mg/mL	0.06	[63]
*G. asiatica*	Fruit	Pakistan	Methanol	Antibacterial	*S. aureus*	MIC was 15.625 µg/mL	0.11	NG	NG	NG	[64]
*G. asiatica*	Bark Fruit	Pakistan	Ethanol	Antibacterial	*S. aureus*	Zone of inhibition 6.33 mm	0.84	Moxifloxacin	Zone of inhibition 30 mm	NG	[65]
*G. asiatica*	Leaves	Pakistan	Methanol	Antibacterial	*S. aureus*	Zone of inhibition 10.4 mm	1.1	Cefixime	Zone of inhibition 20 mm	2.5	[66]
*G. asiatica*	Leaves	Pakistan	Water	Antibacterial	*S. aureus*	Zone of inhibition 13.5 mm	1.6	Cefixime	Zone of inhibition 20 mm	2.5	[66]
*G. optiva*	Leaves	Pakistan	Water	Antibacterial	*S. aureus*	Zone of inhibition 9 mm	0.99	Cephradine	Zone of inhibition 24 mm	1.20	[27]
*G. lasiocarpa*	Stem	South Africa	Chloroform	Antibacterial	*S. aureus*	No activity observed	NG	Streptomycin	Zone of inhibition 12.3 mm	2.31	[15]
*G. hirsuta*	Leaves	India	70% aqueous Methanol	Antibacterial	*S. aureus*	Zone of inhibition 19 mm	0.47	Ciprofloxacin	Zone of inhibition 22 mm	2.16	[67]
*G. asiatica*	Stem Bark	Pakistan	Ethanol	Antibacterial	*S. typhi*	Zone of inhibition 6.33 mm	0.47	Moxifloxacin	Zone of inhibition 19 mm	NG	[65]
*G. asiatica*	Leaves	Pakistan	Methanol	Antibacterial	*S. typhi*	Zone of inhibition 15.2 mm	1.21	Cefixime	Zone of inhibition 21.5 mm	2.58	[66]
*G. asiatica*	Leaves	Pakistan	Water	Antibacterial	*S. typhi*	No activity observed	NG	Cefixime	Zone of inhibition 21.5 mm	2.58	[66]
*G. optiva*	Leaves	Pakistan	Water	Antibacterial	*S. typhi*	Zone of inhibition 10 mm	1.32	Cephradine	Zone of inhibition 21 mm	0.61	[27]
*G. lasiocarpa*	Stem	South Africa	Chloroform	Antibacterial	*S. typhi*	No activity observed	NG	Gentamicin	Zone of inhibition 19.33 mm	1.92	[15]
*G. asiatica*	Leaves	Pakistan	Methanol	Antibacterial	*E. coli*	No activity observed	NG	Cefixime	No activity observed	NG	[66]
*G. asiatica*	Leaves	Pakistan	Water	Antibacterial	*E. coli*	No activity observed	NG	Cefixime	No activity observed	NG	[66]
*G. optiva*	Leaves	Pakistan	Water	Antibacterial	*E. coli*	Zone of inhibition 9 mm	1.20	Cephradine	Zone of inhibition 23 mm	0.20	[27]
*G. lasiocarpa*	Stem	South Africa	Chloroform	Antibacterial	*E. coli*	No activity observed	NG	Gentamicin	Zone of inhibition 18.3 mm	1.68	[15]
*G. hirsuta*	Leaves	India	70% Aqueous methanol	Antibacterial	*E. coli*	Zone of inhibition 16 mm	2.05	Ciprofloxacin	Zone of inhibition 18 mm	0.28	[67]
*G. optiva*	Leaves	Pakistan	Water	Antibacterial	*S. pneumoniae*	Zone of inhibition 10 mm	0.21	Cephradine	Zone of inhibition 25 mm	0.30	[27]
*G. asiatica*	Bark Fruit	Pakistan	Ethanol	Antibacterial	*Proteus vulgaris*	Zone of inhibition 7.33 mm	0.85	Moxifloxacin	Zone of inhibition 16 mm	NG	[65]
*G. asiatica*	Leaves	Pakistan	Ethanol	Antifungal	*Fusarium solani*	MIC was >10 mg/mL	NG	Itraconazole	MIC was 12 mg/mL	0.34	[63]
*G. asiatica*	Fruit	Pakistan	Methanol	Antifungal	*Aspergillus flavus*	Zone of inhibition 35 mm	0.50	NG	NG	NG	[64]
*G. asiatica*	Fruit	Pakistan	Methanol	Antifungal	*Aspergillus niger*	Zone of inhibition 40 mm	0.55	NG	NG	NG	[64]
*G. asiatica*	Fruit	Pakistan	Methanol	Antifungal	*Penicillium notatum*	Zone of inhibition 35 mm	0.90	NG	NG	NG	[64]
*G. asiatica*	Leaves	India	Acetone	Antifungal	*Aspergillus fumigates*	Zone of inhibition 32 mm	0.58	NG	NG	NG	[51]
*G. asiatica*	Leaves	India	Acetone	Antifungal	*Candida glabrata*	Zone of inhibition 28 mm	1.53	NG	NG	NG	[51]
*G. asiatica*	Leaves	India	Acetone	Antifungal	*Aspergillus niger*	Zone of inhibition 25 mm	0.58	NG	NG	NG	[51]
*G. asiatica*	Leaves	Pakistan	Water	Antifungal	*Rhizoctonia solani*	86% inhibition	2	NG	NG	NG	[68]
*G. asiatica*	Leaves	Pakistan	Water	Antifungal	*Fusarium oxysporum*	62% inhibition	1.5	NG	NG	NG	[68]
*G. asiatica*	Leaves	Pakistan	Water	Antifungal	*Macrophomina phaseolina*	81% inhibition	4.1	NG	NG	NG	[68]
Antidiabetic properties of the *Grewia species*											
***Species***	**Plant Part**	**Origin**	**Extraction Solvent**	**Assay**	**Negative** **Control**	**Std Dev**	**Positive Control**	**Std Dev**	**Activity of Extract**	**Std Dev**	**References**
*G. asiatica*	Fruit	Egypt	Ethanol	Rats model	Serum glucose level was 150	10.9	NG	NG	Serum glucose level was 105 at 200 mg/kg of extract	10.4	[69]
*G. asiatica*	Leaf	India	Ethanol	Rats model	Serum glucose level was 227.3	5.9	Serum glucose level was 201 at Glibenclamide 10 mg/kg	6.3	Serum glucose level was 205 at 200 mg/kg of extract	7.1	[70]
*G. asiatica*	Bark	Bangladesh	Ethanol	Rats model	Serum glucose level was 14.9	3	Serum glucose level was 5.9 at Metformin 150 mg/kg	3	Serum glucose level was 7.1	2.5	[66]
*G. asiatica*	Leaf	Pakistan	Methanol	α-Amylase	NG	NG	98% inhibition of α-amylase at Acarbose 0.1 µg/mL	NG	80% inhibition at 500 µg/mL of extract	NG	[71]
*G. asiatica*	Leaf	Pakistan	Methanol	α-Glucosidase	NG	NG	98% inhibition of α-glucosidase at Acarbose 0.1 µg/mL	NG	80% inhibition at 500 µg/mL of extract	NG	[71]
*G. asiatica*	Fruit	India	Aqueous	α-Glucosidase	NG	NG	Acarbose exhibited IC_50_ 0.006 µg/mL in α-glucosidase inhibition	NG	IC_50_ 8.93 mg/mL	NG	[72]
*G. asiatica*	Fruit	India	Aqueous	α-Amylase	NG	NG	Acarbose exhibited IC_50_ 0.83 µg/mL in α-amylase inhibition Inhibition	NG	IC_50_ 0.41 mg/mL	NG	[72]
*G. asiatica*	Pomace	India	20% Hydro-methanol	α-Amylase	NG	NG	IC_50_ 0.39 μg/mL in α-amylase inhibition	NG	IC_50_ 45.7 mg/mL	NG	[30]
*G. asiatica*	Pomace	India	20% Hydro-acetone	α-Amylase	NG	NG	IC_50_ 0.39 μg/mL in α-amylase inhibition	NG	IC_50_ 85.2 mg/mL	NG	[30]
*G. asiatica*	Fruit	Pakistan	Methanol	Non-diabetic human model	NG	NG	NG	NG	1.4% reduction in blood glucose level	NG	[73]

DPPH, 2,2-Diphenyl-1-picrylhydrazyl; FRAP, Ferric reducing antioxidant power; ABTS, 2,2’-azino-bis(3-ethylbenzothiazoline-6-sulfonic acid); NO, Nitric oxide; H_2_O_2_, Hydrogen peroxide; MTT, 3-(4,5-dimethylthiazol-2-yl)-2,5-diphenyl-2H-tetrazolium bromide; NG, Not given activity, and the trend was similar to that revealed in the DPPH and FRAP assays; however, the results cannot be easily compared due to the use of different units.

## Data Availability

Not applicable.

## Supplementary Materials

The following are available online at https://www.mdpi.com/article/10.3390/nu13124565/s1, Supplementary S1. PRISMA checklist. Supplementary S2. Search strategy used in the current review.
