# Supplementary material for: The Chemical Composition and Health-Promoting Effects of the Grewia Species—A Systematic Review and Meta-Analysis
Source: Nutrients. 2021 Dec 20;13(12):4565. doi: 10.3390/nu13124565 (PMC8707743; doi:10.3390/nu13124565)
Supplement: Supplementary file 1 [file nutrients-13-04565-s001.zip › Supplementary S2.pdf]

Supplementary S2. Search strategy used in the current review

Google Scholar, Web of Science, PubMed, Scopus

Genus *Grewia* (e.g., “nutritional composition”, “traditional medicinal uses of *Grewia”,* “biological activities of *genus Grewia”,* “phytochemical composition of *Grewia”*, “antioxidant potential of *Grewia”, “*anticancer analysis of *Grewia”, “in vitro* and *in vivo* anti-inflammatory activities of *Grewia”,* “anti-diabetic properties of *Grewia”*).
